# Wave-function formalism for evolving neural dynamics: a mathematical framework informed by EEG

**DOI:** 10.3389/fncom.2026.1812909

**Published:** 2026-10-12

**Authors:** Madelyn Klebba

**Affiliations:** Independent Researcher, Birmingham, MI, United States

**Keywords:** EEG, epilepsy, neural dynamics, state space (SS) model, wave function

## Abstract

The organization of distributed neural activity remains one of the central challenges in computational neuroscience. Contemporary frameworks, including Integrated Information Theory, Global Workspace Theory, entropy-based approaches, and electromagnetic field theories, have advanced our understanding of neural coordination but employ different mathematical descriptions of large-scale brain dynamics. This study investigates whether wave-function formalism provides a useful mathematical framework for representing evolving neural dynamics measured using electroencephalography (EEG). Rather than introducing a new electrophysiological measure or proposing a new mechanism of consciousness, the framework represents complementary EEG observables within a common state-space description of neural activity. Mathematical inspiration is drawn from Everett’s representation of evolving state spaces, although no claims are made regarding quantum computation or quantum processes in the brain. Electroencephalographic recordings from interictal, preictal, and ictal seizure states (150 EEG segments) were analyzed using complementary measures of signal amplitude, spectral organization, entropy, phase dynamics, cross-frequency coupling, and recurrence quantification. Statistical comparisons employed nonparametric analyses, effect-size estimation, confidence intervals, and *post hoc* testing. Significant differences were observed across all major electrophysiological domains. Ictal recordings exhibited increased signal amplitude, redistribution of spectral power toward lower frequencies, reduced entropy, altered phase organization, changes in cross-frequency coupling, and increased recurrence structure, indicating coordinated changes in evolving neural dynamics during seizure progression. These findings are interpreted within a mathematical framework that treats established EEG observables as complementary descriptors of a single evolving neural state. The principal contribution of this work is the proposal of a mathematically grounded and experimentally testable representation of evolving neural dynamics. Whether this formalism provides new insight into cognition or conscious experience remains an empirical question for future investigation.

## 1 Introduction

### 1.1 Representing evolving neural dynamics

One of the central challenges in contemporary neuroscience is understanding how distributed neural activity evolves through time to produce coherent patterns of brain function. Modern experimental techniques including electroencephalography (EEG), magnetoencephalography (MEG), functional magnetic resonance imaging (fMRI), and intracranial electrophysiology, have demonstrated that neural processing is inherently dynamic, involving continuously changing interactions among spatially distributed neuronal populations. These interactions are reflected in oscillatory activity, phase relationships, large-scale synchronization, and nonlinear temporal dynamics that evolve across multiple spatial and temporal scales.

Although modern neuroscience possesses increasingly sophisticated methods for measuring these dynamics, comparatively less attention has been devoted to the mathematical language used to represent them. Most quantitative EEG analyses examine individual electrophysiological observables including signal amplitude, spectral power, entropy, phase organization, cross-frequency coupling, and recurrence structure, as separate descriptors of neural activity. While each provides valuable information, no single measure captures the full evolution of large-scale neural dynamics. This dissertation investigates whether wave-function formalism provides a useful mathematical framework for representing these evolving neural dynamics. Importantly, the objective is not to propose that the brain is a quantum mechanical system or that neural activity obeys quantum mechanics. Rather, the present work asks whether the mathematical structure of wave-function formalism specifically its representation of evolving complex-valued states, provides a coherent language for describing multiple complementary electrophysiological observables within a single evolving neural-state representation.

The motivation for this investigation arises from one of neuroscience’s enduring questions, how distributed neural activity becomes organized into the coherent stream of thought experienced at any given moment. Existing theories have emphasized large-scale integration, recurrent processing, global broadcasting, and coordinated neural dynamics as essential features of conscious processing (Baars, 1988; Lamme, 2006; Tononi, 2004; Dehaene, 2017). The present work does not attempt to replace these theories. Instead, it asks whether wave-function formalism offers a useful mathematical representation of the neural dynamics they seek to explain.

### 1.2 The problem of multiple concurrent neural configurations

The brain does not operate along a single computational trajectory. At every moment, numerous neural populations simultaneously encode sensory information, memories, predictions, motor plans, attentional processes, and emotional states. Many of these processes occur outside immediate awareness, while others contribute to the unified stream of thought experienced at a given moment. This coexistence of multiple neural processes raises an important representational problem. How should simultaneously evolving neural activity be described mathematically? Conventional electrophysiological analyses quantify individual properties of neural activity but generally do not provide a unified representation of how multiple candidate neural configurations evolve together through time.

The present work therefore introduces the concept of multiple simultaneously active neural configurations as a mathematical description of distributed neural dynamics rather than as independent conscious states. These configurations are intended to represent candidate patterns of coordinated neural activity whose relative contributions evolve continuously within a shared dynamical landscape. Within this framework, the emergence of a unified thought is interpreted as a theoretical consequence of the evolving organization of these neural configurations rather than as an empirical result established directly by the present EEG analyses.

### 1.3 Motivation for wave-function formalism

Throughout the history of science, mathematical formalisms developed within one discipline have frequently found application in others. Fourier analysis, state-space methods, differential equations, and Bayesian inference all originated within particular scientific contexts before becoming fundamental tools across diverse fields. Wave-function formalism represents one such mathematical framework. Its usefulness derives not solely from quantum mechanics but from its ability to describe evolving complex-valued states characterized by changing magnitude and phase. Importantly, adopting this mathematical formalism does not require assuming that the system being described is quantum mechanical. Instead, it provides a compact language for representing systems whose behavior evolves continuously through time.

Electroencephalographic recordings naturally exhibit these properties. The analytic signal obtained through the Hilbert transform provides a complex-valued representation of neural activity whose instantaneous amplitude and phase evolve continuously. The present work therefore investigates whether wave-function formalism provides a useful mathematical language for interpreting this representation and for organizing multiple complementary electrophysiological observables within a common description of evolving neural dynamics. Accordingly, the proposed framework should be understood as a mathematical representation of neural dynamics rather than a physical or ontological theory of consciousness.

### 1.4 Study objectives

The primary objective of this study is to investigate whether wave-function formalism provides a useful mathematical framework for representing evolving neural dynamics measured with EEG. To evaluate this question, EEG recordings obtained during interictal, preictal, and ictal seizure states were analyzed using established electrophysiological measures, including signal amplitude, spectral organization, entropy, phase dynamics, cross-frequency coupling, and recurrence quantification. Rather than introducing new electrophysiological metrics, the study examines whether these established observables evolve in a coordinated manner that can be represented within a common mathematical framework.

The empirical hypotheses evaluated are:

*H1*: Epileptic EEG recordings exhibit greater signal amplitude than non-epileptic recordings.

*H2*: Epileptic EEG recordings exhibit altered entropy relative to non-epileptic recordings.

*H3*: Oscillatory organization differs across interictal, preictal, and ictal brain states.

*H4*: Phase dynamics, cross-frequency coupling, and recurrence characteristics differ across seizure states.

Finally, the study investigates whether the analytic signal,

Ψ(t)=A(t)eiϕ(t)

provides a useful mathematical representation of these coordinated neural dynamics. Building upon this representation, the dissertation proposes a theoretical framework in which multiple simultaneously active neural configurations evolve within a shared neural-state landscape. The implications of this mathematical framework for understanding unified thought are considered in the Discussion, where they are presented as hypotheses and directions for future empirical investigation rather than conclusions established directly by the present EEG analyses.

## 2 Chapter 2

### 2.1 Representing neural dynamics as an evolving state

The human brain is a continuously evolving dynamical system whose activity emerges from the coordinated interactions of billions of neurons distributed across multiple spatial and temporal scales. Rather than operating as a sequence of isolated events, neural activity unfolds through ongoing patterns of oscillation, synchronization, and transient coordination that collectively give rise to measurable electrophysiological signals. Electroencephalography (EEG) provides one of the most direct means of observing these dynamics by recording fluctuations in electrical activity generated by large populations of cortical neurons. Consequently, EEG offers a continuous record of the temporal evolution of large-scale neural activity rather than a static snapshot of isolated neural events (Buzsáki, 2006; Schomer and Lopes da Silva, 2018).

Modern electrophysiology has developed numerous quantitative techniques for characterizing these evolving dynamics. Measures of signal amplitude quantify changes in the magnitude of neural activity, spectral analyses describe the distribution of oscillatory power across frequency bands, entropy measures characterize signal complexity, phase-based metrics capture temporal organization, and recurrence analyses evaluate the evolution of neural trajectories through reconstructed state space (Richman and Moorman, 2000; Bandt and Pompe, 2002; Marwan et al., 2007; Cohen, 2014). Collectively, these methods provide complementary descriptions of neural dynamics, yet they are typically analyzed as independent quantities rather than as components of a unified mathematical representation.

The central premise of the present work is that these diverse electrophysiological observables may instead be interpreted as different properties of a single evolving neural state. This perspective does not replace existing EEG analyses or introduce new electrophysiological measurements. Rather, it proposes a mathematical framework in which multiple established observables are represented within a common formalism describing the continuous evolution of large-scale neural dynamics. In this sense, the contribution of the present work is mathematical rather than electrophysiological. It seeks to provide a unified representational framework for interpreting established EEG observables rather than introducing new measures of neural activity.

A natural mathematical representation of oscillatory systems is provided by the analytic signal obtained through the Hilbert transform. The analytic signal expresses an oscillatory time series as a complex-valued function:

$$\Psi (t)=A(t)e^{\mathrm{i\phi}(t)}$$

Where denotes the instantaneous amplitude of the signal and denotes its instantaneous phase. This representation is widely used throughout signal processing and electrophysiology because it captures two fundamental properties of oscillatory systems, magnitude and temporal organization within a single mathematical object (Le Van Quyen et al., 2001; Cohen, 2014). Importantly, the use of complex-valued notation is entirely classical and should not be interpreted as evidence that the underlying system is quantum mechanical.

The present study adopts this complex-valued representation as the mathematical foundation for describing evolving neural dynamics. The motivation is not to argue that EEG signals obey quantum mechanics or that the brain functions as a quantum system. Rather, motivation is mathematical. Wave-function formalism provides a compact language for representing systems that evolve continuously through time while simultaneously incorporating amplitude, phase, and state evolution within a common framework. Similar mathematical formalisms have frequently found applications outside the scientific domains in which they were originally developed, allowing complex dynamical systems to be represented using common mathematical principles. The present work therefore investigates whether wave-function formalism provides a useful mathematical language for representing large-scale neural dynamics inferred from EEG.

Within this framework, the analytic signal serves as a state representation of the evolving neural system rather than merely a computational tool for extracting instantaneous amplitude and phase. The subsequent sections extend this representation by considering how multiple simultaneously active neural configurations may be described within a shared mathematical state space. This interpretation is introduced as a theoretical framework for organizing distributed neural dynamics and should not be understood as a demonstrated mechanism of consciousness or as a claim regarding the physical ontology of the brain. Instead, the central question explored throughout this dissertation is whether wave-function formalism provides useful mathematical language for representing and interpreting the evolution of large-scale neural dynamics measured using established electrophysiological methods.

### 2.2 Multiple simultaneously active neural configurations

A fundamental principle of modern neuroscience is that neural processing emerges through the coordinated activity of distributed brain networks rather than isolated anatomical regions. Sensory perception, memory retrieval, attentional control, motor planning, emotional processing, and predictive inference are supported by parallel interactions among large-scale neural populations that continuously exchange information across multiple spatial and temporal scales (Hebb, 1949; Edelman, 1989; Buzsáki, 2006; Friston, 2010). Rather than operating independently, these distributed processes are dynamically coordinated through oscillatory synchronization, recurrent interactions, and network-level communication, allowing multiple streams of neural activity to evolve simultaneously (Fries, 2015; Dehaene et al., 1998). The existence of parallel neural processing is therefore well established and provides the biological foundation upon which the present mathematical framework is developed.

The proposed framework does not introduce new neural structures or new electrophysiological mechanisms. Instead, it offers a different mathematical interpretation of how distributed neural activity may be represented. Specifically, the framework proposes that the evolving neural state can be viewed as comprising multiple simultaneously active neural configurations, each representing a transient pattern of coordinated neural activity within the broader dynamical landscape of the brain. These configurations are not intended to represent separate conscious entities, independent minds, or distinct physical systems. Rather, they provide a mathematical description of the multiple interacting patterns of neural activity that coexist within a single biological brain.

A neural configuration is defined here as a transient pattern of coordinated neural dynamics characterized by evolving relationships among amplitude, phase, oscillatory organization, and temporal structure. Such configurations may reflect ongoing sensory processing, memory representations, attentional states, predictive models, or motor preparation, although the present study does not attempt to identify specific cognitive contents. Instead, the concept is introduced as an abstract mathematical representation of distributed neural organization. Multiple neural configurations may coexist at any given moment, continuously evolving as the underlying neural system responds to internal processing and external sensory input. Their relative contributions to the overall neural state change continuously over time, reflecting the inherently dynamic nature of large-scale neural activity.

From this perspective, the objective of the proposed framework is not to explain consciousness directly but to provide mathematical language for representing the evolution of these interacting neural configurations within a common state space. Existing electrophysiological analyses describe individual properties of neural activity, including changes in amplitude, entropy, oscillatory organization, phase relationships, cross-frequency coupling, and recurrence structure. The present framework instead interprets these complementary observables as describing different aspects of a single evolving neural state. The emphasis therefore shifts from analyzing isolated electrophysiological measures to representing the coordinated evolution of distributed neural dynamics as a unified mathematical system.

Importantly, the present EEG analyses do not directly identify individual neural configurations, nor do they demonstrate transitions between them. The single-channel recordings analyzed in this study provide measurements of the temporal organization of neural activity rather than direct observations of distributed cortical interactions. Consequently, the multiple neural configurations proposed here should be understood as components of the mathematical representation rather than empirical entities directly resolved by the present data. Their existence as measurable physiological structures remain a hypothesis for future investigation using multichannel EEG, magnetoencephalography, intracranial recordings, or other techniques capable of resolving large-scale spatial neural dynamics.

Nevertheless, representing neural activity as multiple simultaneously evolving configurations provides a useful conceptual bridge between established electrophysiological observations and the mathematical framework developed in the following sections. The next section introduces wave-function formalism as a compact mathematical representation of this evolving neural state and demonstrates how multiple interacting configurations may be described within a common state-space formalism. The broader implications of this representation for understanding unified thought are considered later in the Discussion, where they are presented as theoretical hypotheses generated by the mathematical framework rather than conclusions established directly by the present EEG analyses.

### 2.3 Wave-function formalism as a state-space representation

Building upon the representation introduced in the previous section, the present framework proposes that large-scale neural dynamics may be interpreted within a common mathematical state space. The objective is not to introduce a new electrophysiological measure or to replace established EEG analyses. Rather, the framework seeks to provide a unified mathematical language in which multiple complementary electrophysiological observables may be interpreted as describing different properties of a single evolving neural state.

The measurable neural state is represented using the analytic signal,

Ψ(t)=A(t)eiϕ(t)

where *A* (*t*), denotes the instantaneous amplitude of the EEG signal and denotes its instantaneous phase. Derived from the Hilbert transform, this representation provides a compact description of oscillatory neural activity by combining two fundamental properties of the signal, magnitude and temporal organization—within a single complex-valued mathematical object (Le Van Quyen et al., 2001; Cohen, 2014). Importantly, the use of complex-valued notation is entirely classical and should not be interpreted as evidence that neural activity is quantum mechanical.

While the analytic signal provides a measurable representation of evolving neural dynamics, it does not specify how the underlying organization of those dynamics should be interpreted. The present framework therefore proposes that the evolving neural state may be viewed mathematically as the superposition of multiple simultaneously active neural configurations,

Ψ(t)=i=1∑Nci(t)Ψi(t)

where Ψ*i* (*t*), denotes the candidate neural configuration and represents its relative contribution to the overall neural state. These coefficients are not interpreted as quantum-mechanical probability amplitudes. Instead, they provide an abstract mathematical description of how multiple distributed patterns of neural activity may collectively contribute to the observable neural state measured using EEG.

Within this framework, the term *superposition* is used in its mathematical sense rather than its quantum-mechanical sense. The representation simply acknowledges that multiple neural processes are known to coexist within the brain and that their collective dynamics may be described within a common state-space formalism. The framework therefore does not propose quantum branching, wave-function collapse, or multiple simultaneously existing conscious observers. Rather, it adopts the mathematical structure of wave-function formalism because it provides a compact representation of systems composed of multiple continuously evolving components. This interpretation is inspired by Everett’s relative-state formulation of quantum mechanics (Everett, 1957; DeWitt and Graham, 1973), but the relationship is strictly mathematical rather than physical. Everett’s formulation demonstrated how multiple evolving configurations could be represented within a single state-space description without requiring collapse into a single physical state. The present framework adopts this representational strategy as a mathematical analogy for describing distributed neural dynamics. No claim is made that the brain obeys quantum mechanics or that neural activity undergoes genuine quantum evolution.

Importantly, the present study does not identify or estimate individual neural configurations directly. The single-channel EEG recordings analyzed in this dissertation provide measurements of the temporal evolution of neural activity rather than direct observations of distributed neural populations. Consequently, the proposed decomposition should be understood as a theoretical representation of the evolving neural state rather than as an experimentally resolved physiological structure. Future investigations employing high-density EEG, magnetoencephalography, intracranial electrophysiology, and computational state-space reconstruction will be required to determine whether candidate neural configurations can be empirically identified and characterized.

The principal contribution of the present framework is therefore mathematical rather than electrophysiological. Established EEG observables, including signal amplitude, entropy, oscillatory organization, phase relationships, cross-frequency coupling, and recurrence dynamics, are interpreted not as isolated measurements but as complementary descriptors of a single evolving neural state. Wave-function formalism provides the mathematical language within which these diverse observables may be represented and interpreted collectively. In this sense, the framework offers a unified state-space representation of neural dynamics while remaining fully compatible with conventional electrophysiological methods.

The implications of this mathematical representation extend beyond the present study. If evolving neural dynamics can be meaningfully represented within this formalism, future work may investigate whether transitions among candidate neural configurations correspond to changes in cognitive processing, attentional selection, or the emergence of unified thought. The present dissertation does not establish these relationships empirically. Rather, it proposes a mathematical framework that generates testable hypotheses for future investigations of distributed neural dynamics.

### 2.4 Stability, metastability, and the evolution of neural states

One of the defining characteristics of large-scale neural systems is their ability to balance stability with flexibility. Neural activity must remain sufficiently organized to support coherent information processing while simultaneously preserving the capacity to adapt rapidly to changing sensory inputs, internal states, and behavioral demands. Contemporary neuroscience increasingly suggests that this balance emerges from the dynamic organization of distributed neural networks rather than from the activity of isolated brain regions (Kelso, 1995; Buzsáki, 2006; Deco et al., 2008; Friston, 2010; Bassett and Sporns, 2017). Functional brain networks therefore exhibit continuous reorganization across multiple spatial and temporal scales, allowing neural processing to remain both coordinated and adaptable (Varela et al., 2001; Fries, 2015).

This balance between persistence and adaptability is commonly described using the concept of metastability. Within dynamical systems theory, metastability refers to the tendency of complex systems to occupy transiently organized configurations while retaining the capacity to reorganize in response to internal dynamics or external perturbations (Kelso, 1995; Friston, 1997; Tognoli and Kelso, 2014). Unlike systems that converge upon a single stable attractor, metastable systems continuously transition among partially coordinated states, allowing simultaneous integration and segregation of neural activity without becoming permanently locked into a single configuration (Tognoli and Kelso, 2008; Tognoli and Kelso, 2014). This perspective has become increasingly influential in systems neuroscience because it provides a theoretical framework capable of explaining how distributed neural populations maintain both coherence and flexibility during ongoing brain function (Buzsáki, 2006; Bassett and Sporns, 2017).

The present framework adopts metastability as a theoretical description of how the proposed mathematical state representation may evolve through time. Within the wave-function formalism introduced in the preceding sections, multiple neural configurations are interpreted as continuously evolving components of a common neural state. Their relative organization changes dynamically as neural activity evolves, allowing the overall neural state to remain coherent while continuously adapting to new sensory information, internal processing, and behavioral demands. Importantly, the present framework does not claim that these neural configurations have been directly identified within the current EEG recordings. Rather, metastability provides a theoretical context within which the proposed mathematical representation may be interpreted (Kelso, 1995; Tognoli and Kelso, 2014).

This interpretation is broadly consistent with the empirical observations reported in the present study. The EEG analyses demonstrated coordinated changes across multiple complementary electrophysiological observables, including signal amplitude, entropy, oscillatory organization, phase relationships, cross-frequency coupling, and recurrence dynamics. Collectively, these measures characterize different aspects of evolving neural dynamics without directly measuring cognition, conscious experience, or thought content. Similar multidimensional approaches have become increasingly common in computational neuroscience because no individual electrophysiological measure fully captures the organization of large-scale neural activity (Le Van Quyen et al., 2001; Cohen, 2014; Marwan et al., 2007). Consequently, the empirical findings should be interpreted as observations of neural-state organization rather than direct evidence for transitions between individual neural configurations.

Pathological transitions, such as epileptic seizures, provide a particularly informative context for examining these dynamics because they involve pronounced alterations in the temporal organization, synchronization, and complexity of neural activity (Lehnertz et al., 2009; Jiruska et al., 2013; Kramer and Cash, 2012; Schomer and Lopes da Silva, 2018). In the present study, reductions in entropy, increases in recurrence structure, alterations in oscillatory organization, and changes in phase relationships were observed during seizure progression. Similar reductions in neural complexity and restrictions in dynamical repertoire have been reported across epilepsy, anesthesia, and disorders of consciousness, suggesting that pathological brain states are often associated with increasingly constrained neural dynamics (Bruhn et al., 2000; Acharya et al., 2015; Mediano et al., 2022). These findings do not demonstrate metastable neural configurations directly, nor do they establish transitions between individual neural configurations. However, they are compatible with the broader hypothesis that pathological brain states involve measurable changes in the flexibility and organization of the evolving neural state.

From this perspective, wave-function formalism should be viewed primarily as a mathematical language for representing the continuous evolution of distributed neural dynamics rather than as a mechanistic theory of consciousness. If future studies employing multichannel EEG, magnetoencephalography, intracranial electrophysiology, and computational state-space reconstruction can identify candidate neural configurations directly, the present framework predicts that their interactions should exhibit metastable organization and continuous temporal evolution. Whether such evolving neural states ultimately prove informative for understanding unified thought remains an empirical question for future investigation. Accordingly, the principal contribution of the present framework is not the demonstration of a mechanism for consciousness, but the proposal of a mathematically coherent state-space representation within which hypotheses concerning neural coordination, cognitive organization, and unified thought may be formulated and experimentally tested. In this sense, metastability provides the theoretical bridge between measurable neural dynamics and the broader research program developed throughout this dissertation.

## 3 Chapter 3: Materials and methods

### 3.1 Overview of the analytical framework

The objective of the present study was to evaluate whether large-scale EEG dynamics associated with epileptic seizure transitions can be represented and interpreted within a wave-function formalism describing evolving neural states. To accomplish this, established electrophysiological signal-processing techniques were applied to segmented EEG recordings obtained from patients with epilepsy. Conventional quantitative EEG analyses were first performed to characterize changes in neural activity across interictal, preictal, and ictal states. The resulting measurements were then interpreted within the dynamical neural-field framework developed in Chapter 2, which proposes that multiple simultaneously active neural configurations evolve within a common neural-field landscape. Importantly, the proposed theoretical neural-field framework did not alter the underlying EEG processing pipeline. Rather, it provided a mathematical and conceptual framework for interpreting measurable properties of large-scale neural dynamics. All signal-processing methods employed in this study are well established within computational neuroscience and clinical EEG research, allowing the proposed theoretical model to be evaluated using conventional electrophysiological analyses rather than introducing novel analytical procedures.

The analytical workflow consisted of several complementary stages. First, EEG recordings were preprocessed to remove signal offsets and prepare the data for subsequent analyses. Frequency-domain characteristics of the recordings were then evaluated using power spectral density (PSD) estimation to quantify dominant oscillatory frequencies and spectral power distributions. Time-domain analyses were subsequently performed to characterize signal amplitude using root-mean-square (RMS) measurements and analytic signal envelopes. To evaluate signal complexity, multiple entropy measures were computed, including spectral entropy, permutation entropy, and sample entropy. These complementary metrics quantify different aspects of neural signal organization and provide estimates of the complexity and predictability of large-scale neural activity (Richman and Moorman, 2000; Bandt and Pompe, 2002). Due to the proposed neural-field framework emphasizing the coordinated interaction of oscillatory activity across multiple temporal scales, the analytic signal of each EEG recording was constructed using the Hilbert transform. Instantaneous amplitude and phase were extracted from the resulting complex-valued representation, allowing quantification of phase organization and cross-frequency interactions. Phase-amplitude coupling (PAC) was evaluated to assess the relationship between slower oscillatory phases and faster oscillatory amplitudes using established modulation-index methods (Le Van Quyen et al., 2001; Canolty and Knight, 2010; Tort et al., 2010).

To further characterize the temporal organization of neural dynamics, recurrence analysis was performed to quantify deterministic structure within the evolving EEG signals. Measures including recurrence rate, determinism, mean diagonal line length, and trajectory speed were computed to evaluate changes in large-scale neural organization across seizure states. Although these analyses do not directly identify metastable neural states, they provide quantitative measures of neural-state organization that are relevant for evaluating the proposed wave-function formalism for representing evolving neural dynamics (Marwan et al., 2007). Finally, statistical analyses were performed to compare electrophysiological measures across interictal, preictal, and ictal recordings. Group differences were evaluated using nonparametric statistical methods, followed by *post hoc* comparisons, effect-size estimation, and confidence intervals to quantify the magnitude and reliability of observed differences. Collectively, these analyses provided the empirical basis for evaluating whether systematic changes in amplitude, entropy, oscillatory organization, cross-frequency coupling, and recurrence dynamics were consistent with the field-theoretic framework proposed in this study.

It is important to emphasize that the analyses presented in this study quantify measurable properties of evolving neural dynamics rather than subjective experience itself. The electrophysiological measures examined including signal amplitude, entropy, oscillatory organization, phase relationships, cross-frequency coupling, and recurrence dynamics, provide complementary descriptions of the temporal organization of neural activity but do not directly identify conscious states, unified thoughts, or the candidate neural configurations proposed in Chapter 2. Accordingly, the empirical results should be interpreted as quantitative observations of neural-state organization rather than direct measurements of consciousness or cognition. The wave-function formalism developed in this dissertation provides a mathematical framework within which these complementary electrophysiological observables may be interpreted collectively as properties of a single evolving neural state. The novelty of the present work therefore lies not in the introduction of new EEG analyses, but in the proposal that established electrophysiological observables can be represented within a unified mathematical formalism. Consequently, the present study evaluates the suitability of this mathematical representation while generating experimentally testable hypotheses for future investigations of distributed neural dynamics and their possible relationship to unified thought.

### 3.2 EEG dataset

The analyses presented in this study were performed using a publicly available electroencephalographic (EEG) dataset consisting of segmented recordings obtained from patients with epilepsy. The dataset was collected through a collaboration between the Neurocomputing Laboratory at the Indian Institute of Technology (IIT) Delhi and the Neurology & Sleep Center, Hauz Khas, New Delhi, India (Swami et al., 2016; Gandhi et al., 2010). The dataset was selected because it contains clearly defined transitions between interictal, preictal, and ictal neural states, providing an appropriate framework for examining changes in large-scale neural dynamics across distinct physiological conditions. The recordings were acquired from 10 patients diagnosed with epilepsy using a Grass Telefactor Comet AS40 Amplification System. Gold-plated scalp electrodes were positioned according to the International 10–20 EEG electrode placement system. Signals were acquired at a sampling frequency of 200 Hz and hardware filtered between 0.5 and 70 Hz during data collection. These acquisition parameters are consistent with conventional clinical EEG recording practices and provide adequate temporal resolution for evaluating oscillatory dynamics across multiple frequency bands.

The dataset was organized into three clinically relevant classes representing different stages of epileptic activity:

- Interictal: EEG recordings obtained between seizure events, representing the baseline pathological state.
- Preictal: EEG recordings immediately preceding seizure onset, representing the transition toward ictal activity.
- Ictal: EEG recordings acquired during active epileptic seizures.

Each class contained 50 EEG segments, yielding a total of 150 recordings for analysis. Every segment consisted of 1,024 samples, corresponding to 5.12 s of continuous EEG activity. Each recording was stored as an individual MATLAB (*.mat*) file containing a single-channel EEG time series. The relatively short duration of each recording minimizes long-term non-stationarity while preserving the oscillatory characteristics associated with each seizure state. All recordings were acquired using identical instrumentation and acquisition parameters, comparisons among interictal, preictal, and ictal conditions were not confounded by differences in hardware configuration or sampling methodology.

The present investigation did not attempt to classify seizure activity or develop a diagnostic algorithm. Instead, the dataset was used as a model system for examining whether measurable properties of large-scale neural dynamics, including amplitude, spectral organization, entropy, phase relationships, cross-frequency coupling, and recurrence characteristics, change systematically across distinct seizure states. Within the dynamical neural-field framework proposed in this dissertation, these electrophysiological measurements provide quantitative descriptors of neural-field organization rather than direct measurements of conscious experience. This dataset has been extensively used in the development and evaluation of automated seizure-detection algorithms, it provides a well-characterized benchmark for quantitative EEG analysis (Swami et al., 2016; Gandhi et al., 2010). Employing an established public dataset improves reproducibility and allows future investigations to compare the dynamical neural-field framework proposed here with alternative computational approaches using identical electrophysiological recordings.

### 3.3 Complex-valued state representation

To characterize the instantaneous dynamics of each EEG recording, the neural signal was represented using the analytic signal obtained through the Hilbert transform. The analytic signal provides a complex-valued representation of an oscillatory time series in which instantaneous amplitude and phase can be estimated continuously over time (Le Van Quyen et al., 2001). This representation provides a mathematically rigorous description of the temporal evolution of electrophysiological activity while remaining entirely within the domain of conventional signal processing.

For each EEG segment, the analytic signal was expressed as

$$\Psi (t)=A(t)e^{\mathrm{i\phi}(t)}$$

where A(t) represents the instantaneous amplitude of the neural signal, ϕ(t) represents its instantaneous phase, and $i=\sqrt{-1}$ is the imaginary unit. Instantaneous amplitude was computed as the magnitude of the analytic signal, whereas instantaneous phase was obtained from its complex argument. Throughout the present study, this measurable representation formed the basis for quantifying amplitude dynamics, phase organization, and cross-frequency interactions using established electrophysiological methods.

Importantly, the use of the analytic signal should not be interpreted as evidence that neural activity is fundamentally quantum mechanical. The complex-valued representation employed here is a standard mathematical tool widely used in electrophysiology and signal processing to describe oscillatory dynamics (Le Van Quyen et al., 2001; Cohen, 2014). In the present framework, the analytic signal serves as an empirical representation of large-scale neural dynamics rather than a quantum-mechanical wave function.

While the analytic signal describes the observable neural field, it does not specify the internal organization responsible for its evolution. The theoretical framework developed in Chapter 2 therefore proposes that the macroscopic neural field may be conceptualized as the collective interaction of multiple simultaneously active neural configurations,

$$\Psi_{\text{field}}(t)=\sum_{i=1}^{N}c_{i}(t)\Psi_{i}(t)$$

where $\Psi_{i}(t)$ represents the $i^{\mathrm{th}}$ neural configuration and $c_{i}(t)$ represents its relative dynamical contribution to the overall neural-field landscape. These coefficients should not be interpreted as probabilities or quantum amplitudes, but rather as abstract quantities describing the relative influence of distributed neural configurations within the evolving neural field.

The proposed framework further suggests that unified thought corresponds to the transient dominance of one neural configuration within this evolving landscape. Conceptually, this process is represented as

$$\Psi_{\text{thought}}(t)=\underset{i}{\arg\;\max}C_{i}(t)$$

where $C_{i}(t)$ denotes a theoretical neural coordination functional describing the relative organization of each neural configuration. The operator identifies the configuration exhibiting the greatest degree of coordinated neural organization at a given moment while alternative configurations remain dynamically active within the broader neural field. This representation does not imply that competing neural configurations disappear; rather, it proposes that one configuration temporarily dominates the macroscopic organization of the neural field and corresponds to the current unified thought.

The neural coordination functional is proposed conceptually as

$$C_{i}(t)=f(A_{i},\phi_{i},H_{i},P,A,C_{i}\mathrm{RQ}A_{i}\ldots )$$

where $A_{i}$ represents signal amplitude, $\phi_{i}$ phase organization, $H_{i}$ entropy, ${\mathrm{PAC}}_{i}$ phase-amplitude coupling, and $\mathrm{RQ}A_{i}$ recurrence quantification measures associated with the $i^{\mathrm{th}}$ neural configuration. These variables are not assumed to contribute equally, nor is the explicit functional form specified in the present study. Rather, this formulation provides a theoretical framework for integrating multiple complementary electrophysiological observables into a common description of large-scale neural organization.

Importantly, neither the individual neural configurations nor the neural coordination functional are directly estimated from the EEG recordings analyzed in this dissertation. The empirical analyses are performed entirely using measurable electrophysiological quantities derived from the analytic signal, including amplitude, spectral organization, entropy, phase dynamics, cross-frequency coupling, and recurrence characteristics. The theoretical decomposition presented here therefore serves as an interpretive framework for understanding the organization of large-scale neural dynamics rather than a direct model fitted to the observed data.

This distinction between empirical measurement and theoretical interpretation is fundamental to the present study. The EEG analyses quantify objective properties of large-scale neural dynamics, whereas the proposed dynamical neural-field framework provides one possible mathematical description of how these measurable dynamics may contribute to the emergence of unified thought through transient neural-field dominance.

### 3.4 Frequency- and time-domain analyses

The electrophysiological characteristics of each EEG recording were evaluated using complementary frequency-domain and time-domain analyses. These analyses were selected to quantify oscillatory activity, signal power, and temporal amplitude dynamics across interictal, preictal, and ictal seizure states using established methods in quantitative EEG research.

#### 3.4.1 Frequency-domain analysis

The spectral composition of each EEG segment was examined using power spectral density (PSD) estimation computed with Welch’s (1967) averaged periodogram method. Welch’s method was selected because it provides a robust estimate of spectral power while reducing the variance associated with conventional periodogram estimation through windowing and segment averaging. Power spectral density estimates were calculated for each EEG recording to characterize the distribution of oscillatory power across frequencies. From these spectra, dominant oscillatory frequencies and relative spectral power were quantified. Frequency-domain analysis was used to evaluate whether seizure progression was associated with systematic redistribution of oscillatory energy across the EEG spectrum.

To further characterize neural oscillatory activity, spectral power was examined within the conventional EEG frequency bands:

- Delta (0.5–4 Hz).
- Theta (4–8 Hz).
- Alpha (8–13 Hz).
- Beta (13–30 Hz).
- Gamma (30–70 Hz).

These frequency bands represent well-established functional divisions of EEG activity and provide a standardized framework for comparing oscillatory dynamics across physiological and pathological brain states (Buzsáki and Draguhn, 2004; Cohen, 2014).

#### 3.4.2 Time-domain analysis

Time-domain analyses were performed to quantify changes in signal amplitude associated with seizure activity. Because epileptic seizures are frequently characterized by increases in synchronized neuronal firing, amplitude-based measures provide important indicators of large-scale neural activation. Signal amplitude was quantified using the root-mean-square (RMS) value of each EEG segment. RMS provides a stable estimate of the overall signal magnitude by incorporating both positive and negative voltage fluctuations into a single measure of electrical activity. Compared with peak amplitude measurements, RMS is less sensitive to isolated transient events and provides a more representative estimate of average neural activation over the duration of the recording.

In addition to RMS, the instantaneous amplitude envelope of each EEG recording was obtained from the analytic signal generated by the Hilbert transform. The amplitude envelope provides a continuous estimate of changes in oscillatory magnitude over time and allows visualization of transient increases in neural activity that may accompany seizure evolution (Le Van Quyen et al., 2001). Both RMS amplitude and analytic signal envelopes were computed for every EEG recording. Mean amplitude values were subsequently compared across interictal, preictal, and ictal conditions to determine whether seizure progression was associated with systematic changes in large-scale neural activation. The frequency-domain and time-domain analyses presented in this section constitute conventional quantitative EEG measurements and do not, by themselves, provide evidence regarding conscious processing. Rather, these analyses quantify measurable properties of neural activity that are subsequently interpreted within the dynamical neural-field framework proposed in this dissertation.

### 3.5 Entropy analysis

To quantify changes in the complexity and organization of neural activity across seizure states, multiple entropy measures were computed for each EEG recording. Entropy-based analyses provide quantitative estimates of the irregularity, unpredictability, and information content of physiological time series and have become widely used for characterizing changes in neural dynamics associated with both normal and pathological brain activity (Richman and Moorman, 2000; Bandt and Pompe, 2002). With no single entropy measure completely characterizing the complexity of EEG signals, three complementary entropy metrics were evaluated: spectral entropy, permutation entropy, and sample entropy. Together, these measures provide a multidimensional assessment of neural signal organization by quantifying different aspects of temporal and spectral complexity.

#### 3.5.1 Spectral entropy

Spectral entropy was calculated from the normalized power spectral density (PSD) of each EEG recording. Spectral entropy applies Shannon’s information entropy to the frequency-domain representation of a signal, providing a quantitative measure of how oscillatory power is distributed across the EEG spectrum (Shannon, 1948; Inouye et al., 1991). Higher spectral entropy values indicate a broader and more uniform distribution of spectral power across frequencies, whereas lower values indicate increasing concentration of oscillatory activity within a limited number of dominant frequencies. In the context of EEG analysis, reductions in spectral entropy have been associated with increased neural synchronization and reduced complexity in a variety of physiological and pathological states (Inouye et al., 1991; Sabeti et al., 2009). Within the present study, spectral entropy was used as an objective measure of large-scale spectral organization and was compared across interictal, preictal, and ictal seizure states to evaluate whether seizure progression was accompanied by systematic changes in the distribution of oscillatory activity.

#### 3.5.2 Permutation entropy

Permutation entropy was computed to evaluate the temporal complexity of the EEG recordings based on the ordinal relationships among successive data points (Bandt and Pompe, 2002). Unlike traditional statistical measures, permutation entropy is relatively insensitive to changes in signal amplitude and instead quantifies the diversity of temporal patterns within the time series. Lower permutation entropy values indicate greater temporal regularity and increased predictability of the signal, whereas higher values reflect greater temporal complexity. This measure has been widely applied to EEG because of its computational efficiency and robustness in the presence of physiological noise.

#### 3.5.3 Sample entropy

Sample entropy was calculated to quantify the regularity of the EEG time series by estimating the likelihood that similar patterns of neural activity remain similar over time (Richman and Moorman, 2000). Compared with approximate entropy, sample entropy provides a less biased estimate of signal regularity and is less sensitive to recording length. Lower sample entropy values indicate increased regularity and reduced complexity of neural activity, whereas higher values indicate greater irregularity and dynamical variability. Because seizure activity often involves increased synchronization and reduced variability of neuronal firing, sample entropy provides a complementary measure of changes in large-scale neural organization.

#### 3.5.4 Interpretation of entropy measures

The entropy measures employed in this study were selected because each characterizes a distinct aspect of neural signal complexity. Spectral entropy evaluates the distribution of oscillatory power across frequencies, permutation entropy characterizes the diversity of temporal ordering within the signal, and sample entropy quantifies the predictability of neural activity over time. Evaluating these complementary measures together provides a more comprehensive description of large-scale neural organization than any single entropy metric alone. Within the present investigation, entropy analyses were used exclusively as quantitative descriptors of electrophysiological signal organization. These measures do not constitute direct measurements of conscious awareness or cognitive state. Rather, they provide objective indices of neural complexity that were subsequently compared across interictal, preictal, and ictal recordings. Any interpretation of these findings within the proposed dynamical neural-field framework is therefore presented as a theoretical explanation of the observed neural dynamics rather than as a direct consequence of the entropy measures themselves.

### 3.6 Phase organization and cross-frequency coupling

In addition to quantifying amplitude and signal complexity, the temporal organization of neural oscillations was evaluated using measures of instantaneous phase and cross-frequency coupling. These analyses provide information regarding the coordination of neural activity across multiple temporal scales and have become widely used for investigating large-scale communication within the brain (Canolty and Knight, 2010; Cohen, 2014).

#### 3.6.1 Instantaneous phase estimation

Instantaneous phase was estimated from the analytic signal obtained using the Hilbert transform. The Hilbert transform provides a complex-valued representation of the EEG signal from which both instantaneous amplitude and phase can be extracted continuously over time (Le Van Quyen et al., 2001). For each EEG segment, instantaneous phase was calculated as the angular component of the analytic signal,

[\phi(t)=\arg\left[\Psi(t)\right]]

where (\Psi (*t*)) denotes the analytic signal and (\arg (\cdot)) represents its complex phase angle.

Phase information was used to characterize the temporal organization of oscillatory activity rather than absolute signal magnitude. Unlike amplitude-based measures, phase estimates describe the timing relationships of neural oscillations and provide a basis for evaluating coordination among rhythmic processes.

#### 3.6.2 Phase-amplitude coupling

Cross-frequency interactions were quantified using phase-amplitude coupling (PAC). PAC measures the degree to which the phase of a lower-frequency oscillation systematically modulates the amplitude of a higher-frequency oscillation, providing an estimate of coordination across temporal scales (Canolty and Knight, 2010; Tort et al., 2010). Within the present study, phase-amplitude coupling was quantified using the Modulation Index (MI) described by Tort et al. (2010). The phase of the theta frequency band (4–8 Hz) was coupled with the amplitude envelope of the gamma frequency band (30–70 Hz) to evaluate interactions between slower coordinating oscillations and faster local neural activity. Theta–gamma coupling was selected because it represents one of the most extensively studied forms of cross-frequency interaction and has been associated with large-scale neural coordination in both normal and pathological brain states (Canolty and Knight, 2010; Tort et al., 2010). Prior to PAC estimation, the EEG recordings were band-pass filtered into the respective theta and gamma frequency ranges. Instantaneous phase and amplitude were extracted using the Hilbert transform, after which the Modulation Index was calculated to quantify the degree to which gamma-band amplitude was systematically modulated by theta-band phase.

To reduce the likelihood of spurious coupling arising from finite data length or signal autocorrelation, surrogate analyses were performed by generating randomized phase relationships while preserving the spectral properties of the original recordings. Observed modulation indices were subsequently compared with the surrogate distributions to determine whether measured coupling exceeded values expected by chance (Tort et al., 2010).

#### 3.6.3 Interpretation of phase measures

The phase-based analyses employed in this study were intended to characterize the temporal organization of neural oscillations rather than to identify specific cognitive or conscious states. Instantaneous phase and cross-frequency coupling quantify measurable electrophysiological relationships among oscillatory processes and therefore provide complementary information to amplitude- and entropy-based analyses. Within the proposed field-theoretic framework, changes in phase organization and cross-frequency coupling are interpreted as quantitative descriptors of large-scale neural coordination. However, the empirical analyses themselves do not directly measure neural-field configurations or conscious awareness. Instead, they provide objective measures of oscillatory organization that may subsequently be interpreted within the theoretical framework developed in Chapter 2.

### 3.7 Recurrence analysis

To further characterize the temporal organization of neural dynamics, recurrence analysis was performed on each EEG recording. Recurrence analysis is a nonlinear time-series technique that quantifies the extent to which a dynamical system revisits previously occupied regions of its state space (Eckmann et al., 1987; Marwan et al., 2007). Unlike conventional spectral analyses, recurrence methods evaluate the temporal structure of signal evolution and are therefore well suited for investigating changes in neural dynamics associated with transitions between physiological and pathological brain states.

#### 3.7.1 State-space reconstruction

Prior to recurrence analysis, each EEG segment was reconstructed in state space using time-delay embedding according to Takens’ embedding theorem (Takens, 1981). State-space reconstruction transforms the one-dimensional EEG time series into a multidimensional representation that preserves the temporal evolution of the underlying dynamics. State-space reconstruction was performed using a fixed embedding dimension of m=3 and a time delay of τ=8 samples, corresponding to 40 ms at the 200 Hz sampling rate. Each EEG segment was *z*-scored prior to embedding. Reconstructed state vectors were compared using Euclidean distance, and the recurrence threshold was selected separately for each segment to produce a fixed recurrence density of 10%. Diagonal line structures shorter than two samples were excluded from determinism and mean diagonal length calculations.

X(t)=[x(t),,x(t+τ),,x(t+2τ)]

where m=3 and τ=8 samples.

#### 3.7.2 Recurrence quantification analysis

Following state-space reconstruction, recurrence plots were generated for each EEG segment. Recurrence plots provide a graphical representation of repeated visits to similar regions of state space and form the basis for Recurrence Quantification Analysis (RQA), which summarizes dynamical organization using quantitative measures (Marwan et al., 2007).

The following RQA metrics were computed for each recording:

- Recurrence rate (RR): The proportion of recurrent points within the recurrence plot, representing the overall frequency with which the neural system revisits previously occupied states.
- Determinism (DET): The proportion of recurrent points forming diagonal line structures, reflecting the degree to which neural dynamics follow predictable temporal trajectories.
- Mean diagonal line length (L): The average length of diagonal structures within the recurrence plot, providing an estimate of the persistence of recurrent dynamical patterns.
- Trajectory speed: An estimate of the rate at which the reconstructed neural trajectory evolves through state space, reflecting changes in the temporal progression of neural dynamics.

Together, these measures provide complementary descriptions of the organization, regularity, and temporal evolution of the reconstructed neural system.

#### 3.7.3 Interpretation of recurrence measures

Recurrence analysis was used in the present study to characterize the dynamical organization of EEG activity rather than to identify specific neural mechanisms or conscious states. Changes in recurrence structure reflect measurable alterations in the temporal evolution of large-scale neural dynamics and therefore complement the amplitude, spectra, entropy, and phase-based analyses described in the preceding sections. Within the proposed dynamical neural-field framework, recurrence measures provide quantitative descriptors of how neural activity evolves over time. Increased recurrence or determinism may indicate greater persistence of particular dynamical patterns, whereas reduced recurrence may reflect increased variability of neural activity. These measurements should not be interpreted as direct evidence of metastable neural-field configurations; rather, they provide objective indices of neural dynamics that may subsequently be interpreted within the broader theoretical framework. The principal outcome measures obtained from the recurrence analysis were Recurrence Rate (RR), Determinism (DET), Mean Diagonal Line Length (L), and Trajectory Speed, each of which was statistically compared across interictal, preictal, and ictal seizure states.

### 3.8 Statistical analysis

Statistical analyses were performed to evaluate differences in electrophysiological measures among interictal, preictal, and ictal EEG recordings. Because several of the measured variables exhibited departures from normality and the dataset consisted of independent groups of equal size, nonparametric statistical methods were selected to provide robust comparisons across seizure states.

#### 3.8.1 Overall group comparisons

For each electrophysiological measure, overall differences among the three seizure states were evaluated using the Kruskal–Wallis *H* test, the nonparametric analog of one-way analysis of variance (Kruskal and Wallis, 1952). The Kruskal–Wallis test determines whether one or more groups differ significantly in their median values without assuming normally distributed data.

The Kruskal–Wallis test was applied independently to all primary outcome variables, including:

- Root-mean-square (RMS) amplitude.
- Dominant frequency.
- Relative spectral power.
- Spectral entropy.
- Permutation entropy.
- Sample entropy.
- Alpha-band temporal phase concentration.
- Theta–gamma phase-amplitude coupling (Modulation Index).
- Recurrence Rate (RR).
- Determinism (DET).
- Mean diagonal line length.
- Mean state-space displacement.

Statistical significance was evaluated using a two-sided significance level of *α* = 0.05.

#### 3.8.2 Pairwise *post hoc* comparisons

When significant overall group differences were identified, pairwise comparisons between seizure states were performed using the Mann–Whitney U test. Because multiple pairwise comparisons were conducted for each electrophysiological measure, *p*-values were adjusted using the Holm correction to control the family-wise error rate while maintaining greater statistical power than the traditional Bonferroni procedure (Holm, 1979).

The following comparisons were evaluated for each significant outcome measure:

- Interictal vs. Preictal.
- Interictal vs. Ictal.
- Preictal vs. Ictal.

Both uncorrected and Holm-adjusted *p*-values are reported in the Results chapter.

#### 3.8.3 Effect size estimation

Statistical significance alone does not quantify the magnitude of observed differences. Accordingly, effect sizes were calculated for both overall and pairwise comparisons. For the Kruskal–Wallis analyses, epsilon-squared (*ε*^2^) was calculated as a measure of the proportion of variability attributable to seizure state. Pairwise comparisons were further characterized using Cohen’s *d*, providing a standardized estimate of the magnitude of differences between seizure conditions.

#### 3.8.4 Confidence intervals

To quantify the precision of estimated group means, 95% bootstrap confidence intervals were computed for each electrophysiological measure. Bootstrap confidence intervals were generated using 5,000 resampling iterations with replacement, providing robust interval estimates without assuming normally distributed data (Efron and Tibshirani, 1993). Mean values, standard deviations, medians, and corresponding confidence intervals are reported for each seizure state to facilitate comparison of neural dynamics across interictal, preictal, and ictal recordings.

#### 3.8.5 Statistical software

All analyses were performed using Python. Numerical computations were conducted using NumPy, signal-processing procedures were implemented using SciPy, statistical analyses were performed using SciPy and statsmodels, and tabular data management was conducted using Pandas. Figures were generated using Matplotlib. All statistical procedures were implemented within a fully reproducible analysis pipeline developed specifically for the present study, allowing every reported metric, statistical comparison, and figure to be regenerated directly from the original EEG recordings.

#### 3.8.6 Interpretation of statistical results

The statistical analyses described in this chapter were designed to evaluate measurable differences in electrophysiological properties across interictal, preictal, and ictal EEG recordings. These analyses quantify changes in signal amplitude, oscillatory organization, entropy, phase dynamics, cross-frequency coupling, and recurrence structure using established statistical methods. They do not directly identify candidate neural configurations, measure subjective experience, or establish mechanisms of consciousness or unified thought.

Accordingly, statistically significant differences should be interpreted as quantitative descriptions of evolving neural dynamics rather than direct validation of the theoretical framework proposed in Chapter 2. The wave-function formalism developed in this dissertation provides a mathematical language within which these complementary electrophysiological observables may be represented collectively as properties of a single evolving neural state. The principal contribution of the present work is therefore not the introduction of new electrophysiological measures, but the proposal that established EEG observables can be interpreted within a unified mathematical state-space representation. The statistical results presented in Chapters 3 and 4 provide the empirical foundation for evaluating this representational framework and for generating experimentally testable hypotheses that can be investigated in future studies of distributed neural dynamics and neural-state organization.

## 4 Results

### 4.1 Overview of cohort results

A total of 150 electroencephalographic (EEG) recordings were analyzed, comprising 50 interictal, 50 preictal, and 50 ictal recordings obtained from 10 patients with epilepsy. Each recording consisted of a single-channel EEG segment containing 1,024 samples, corresponding to 5.12 s of continuous neural activity sampled at 200 Hz. All recordings were successfully processed using the analysis pipeline described in Chapter 3, and no files were excluded from the final analyses. The analytical pipeline quantified complementary electrophysiological characteristics of each recording, including signal amplitude, spectral composition, entropy, temporal phase organization, cross-frequency coupling, and recurrence dynamics. For each metric, overall differences among seizure states were evaluated using the Kruskal–Wallis *H* test, followed by Holm-corrected pairwise Mann–Whitney *U* tests when appropriate. Effect sizes were quantified using epsilon-squared (*ε*^2^) for overall group comparisons and Cohen’s *d* for pairwise comparisons. In addition, 95% bootstrap confidence intervals were calculated for all primary outcome measures.

Across the cohort, statistically significant differences were identified for multiple electrophysiological measures, including root-mean-square (RMS) amplitude, spectral entropy, permutation entropy, sample entropy, alpha-band temporal phase concentration, theta-gamma phase-amplitude coupling, recurrence determinism, and mean state-space displacement. Dominant frequency also differed significantly among seizure states, although the corresponding effect size was comparatively small. Complete statistical results for each electrophysiological measure are presented in the following sections.

The results presented in this chapter describe quantitative differences in established electrophysiological measures across interictal, preictal, and ictal EEG recordings. These findings characterize changes in evolving neural dynamics but should not be interpreted as direct measurements of candidate neural configurations, unified thought, or conscious experience. Their interpretation within the mathematical framework developed in Chapters 1 and 2 is reserved for the Discussion (see Table 1).

**Table 1:** Summary of primary electrophysiological findings across seizure states.

Analysis	Primary outcome	*H*	*p*	*ε* ^2^	Magnitude of effect	Principal observation
RMS amplitude	Overall signal magnitude	98.68	<0.001	0.658	Very large	Progressive increase in EEG amplitude from interictal to ictal recordings
Hilbert envelope	Instantaneous signal amplitude	98.65	<0.001	0.658	Very large	Strong increase in envelope amplitude during seizures
Dominant frequency	Peak oscillatory frequency	7.23	0.027	0.036	Small	Shift toward slower dominant frequencies during seizures
Delta relative power	Relative spectral power	70.06	<0.001	0.463	Large	Progressive increase in delta-band dominance
Theta relative power	Relative spectral power	85.85	<0.001	0.570	Very large	Largest redistribution of oscillatory power across seizure states
Alpha relative power	Relative spectral power	66.50	<0.001	0.439	Large	Progressive reduction in alpha activity
Beta relative power	Relative spectral power	6.20	0.045	0.029	Small	Minimal change across seizure states
Gamma relative power	Relative spectral power	43.59	<0.001	0.283	Moderate	Reduced gamma contribution during seizures
Spectral entropy	Spectral complexity	72.49	<0.001	0.480	Large	Decreased spectral complexity during seizure progression
Permutation entropy	Temporal complexity	41.35	<0.001	0.268	Moderate	Reduced temporal complexity across seizure states
Sample entropy	Signal irregularity	88.16	<0.001	0.586	Very large	Marked reduction in signal complexity during seizures
Alpha-phase temporal concentration	Temporal phase organization	22.49	<0.001	0.139	Small–moderate	Altered temporal phase organization during seizures
Theta–gamma PAC	Cross-frequency coupling	29.25	<0.001	0.185	Moderate	Significant alterations in theta–gamma coupling
Recurrence rate (RR)	State-space recurrence	76.49	<0.001	0.507	Large	Consistent differences in recurrence structure among seizure states
Determinism (DET)	Temporal predictability	38.09	<0.001	0.246	Moderate	Increased persistence of dynamical trajectories during seizures
Mean diagonal line length	Persistence of recurrent trajectories	90.55	<0.001	0.602	Very large	Longer recurrent trajectory segments during ictal activity
Mean state-space displacement	State-space evolution	65.23	<0.001	0.430	Large	Reduced movement through state space during seizure progression

*H*, Kruskal–Wallis test statistic; *ε*^2^, effect size estimate for the Kruskal–Wallis test. Magnitude of effect was interpreted qualitatively using conventional thresholds, with larger *ε*^2^ values indicating greater separation among seizure states. “Principal Observation” provides a concise descriptive summary of the observed electrophysiological changes and is not intended as a mechanistic interpretation.

### 4.2 Frequency- and time-domain results

Descriptive statistics are shown in (see Tables 2, 3) and (see Figures 1, 2):

**Table 2:** Descriptive statistics for frequency- and time-domain measures.

Measure	Interictal (mean ± SD)	95% CI	Preictal (mean ± SD)	95% CI	Ictal (mean ± SD)	95% CI
RMS amplitude	23.49 ± 7.44	21.43–25.61	29.37 ± 18.20	24.45–34.44	138.94 ± 49.85	126.16–153.39
Hilbert envelope	28.79 ± 9.16	26.28–31.42	34.16 ± 19.72	28.78–39.60	179.00 ± 67.59	161.67–198.70
Dominant frequency (Hz)	4.70 ± 3.09	3.88–5.58	3.63 ± 2.87	2.89–4.48	2.59 ± 0.43	2.47–2.72

Values are reported as mean ± standard deviation. CI, 95% bootstrap confidence interval.

**Table 3:** Overall statistical comparisons for frequency- and time-domain measures.

Measure	Kruskal–Wallis *H*	*p*	*ε* ^2^
RMS amplitude	98.68	<0.001	0.658
Hilbert envelope	98.54	<0.001	0.657
Dominant frequency	7.23	0.027	0.036

*ε*^2^ represents the Kruskal–Wallis effect size. Larger values indicate a greater proportion of variance attributable to seizure state.

**Figure 1:**
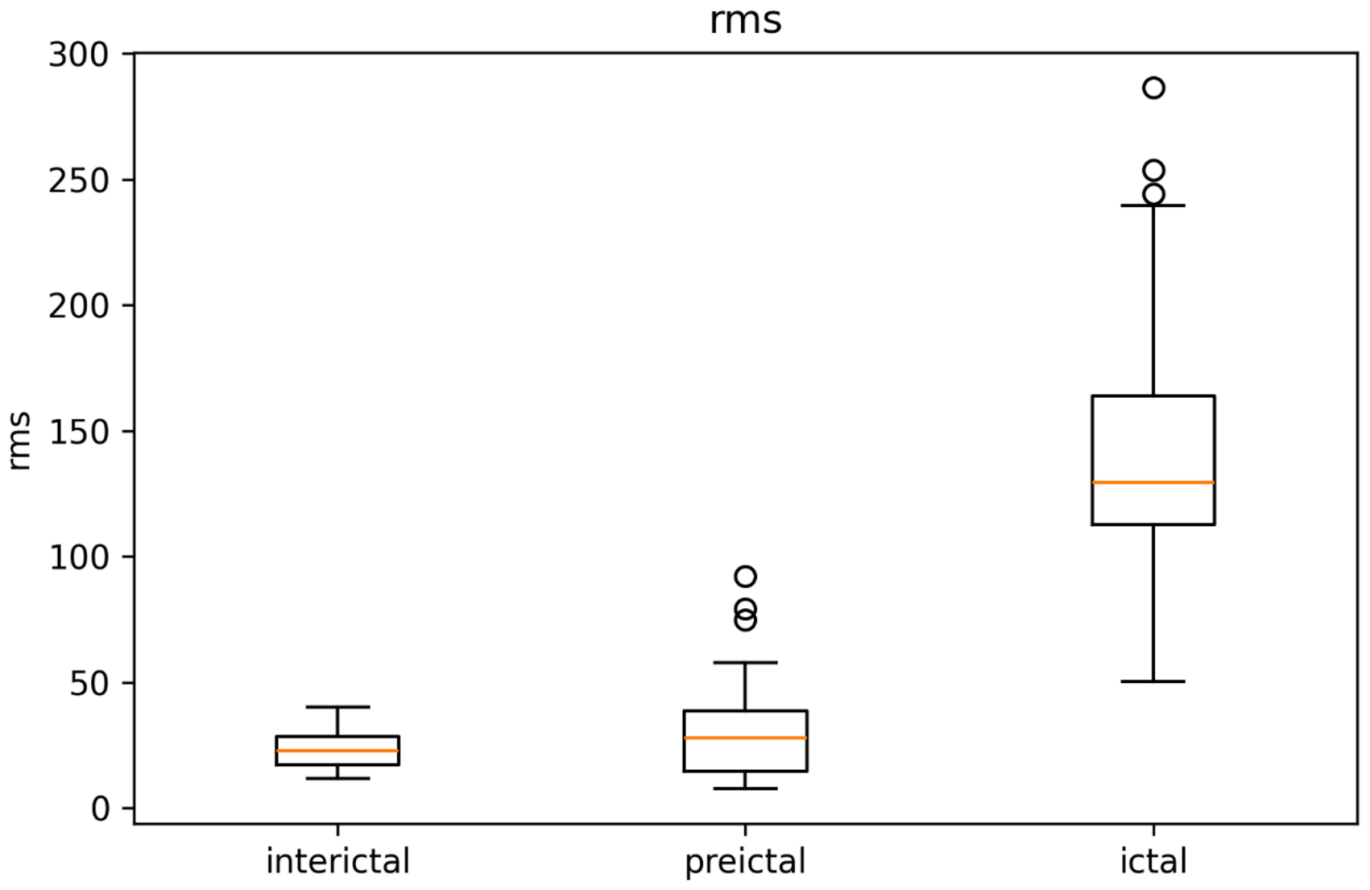
RMS amplitude across seizure states. Distribution of root-mean-square (RMS) amplitude for interictal, preictal, and ictal EEG recordings. Boxes represent the interquartile range, horizontal lines indicate the median, whiskers denote the range excluding outliers, and individual points represent recordings.

**Figure 2:**
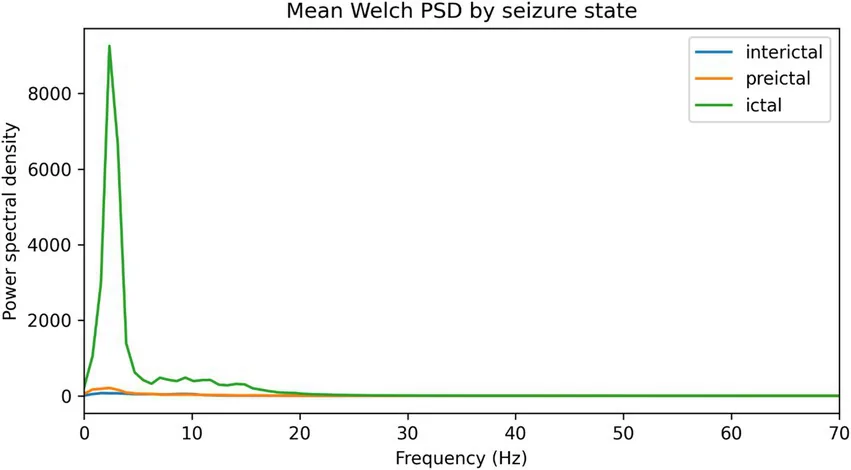
Mean power spectral density. Mean Welch power spectral density for interictal, preictal, and ictal EEG recordings. Curves represent the average spectral power across recordings within each seizure state.

#### 4.2.1 Results

Root-mean-square (RMS) amplitude differed significantly among seizure states (*H* = 98.68, *p* < 0.001, *ε*^2^ = 0.658), representing the largest effect observed among the frequency- and time-domain measures. Mean RMS amplitude increased from 23.49 ± 7.44 μV during the interictal state to 29.37 ± 18.20 μV during the preictal state and 138.94 ± 49.85 μV during ictal recordings, indicating a substantial increase in overall EEG signal magnitude during seizure activity.

A similar pattern was observed for the Hilbert envelope, which provides a measure of instantaneous signal amplitude. Mean envelope magnitude increased progressively across seizure states, from 28.79 ± 9.16 in interictal recordings to 34.16 ± 19.72 in preictal recordings and 179.00 ± 67.59 during ictal recordings. The corresponding overall group comparison also demonstrated a large effect (*H* = 98.54, *p* < 0.001, *ε*^2^ = 0.657), consistent with marked increases in signal energy during seizure onset.

Dominant oscillatory frequency also differed significantly among seizure states (*H* = 7.23, *p* = 0.027, *ε*^2^ = 0.036), although the magnitude of this effect was comparatively small. Mean dominant frequency decreased from 4.70 Hz during interictal recordings to 3.63 Hz during preictal recordings and 2.59 Hz during ictal recordings, indicating a shift toward slower oscillatory activity during seizure evolution. Collectively, these findings demonstrate that seizure progression was characterized primarily by pronounced increases in signal amplitude and a modest reduction in dominant oscillatory frequency (see Table 4).

**Table 4:** Descriptive statistics for relative spectral power.

Frequency band	Interictal	Preictal	Ictal
Delta	0.400 ± 0.104	0.514 ± 0.192	0.754 ± 0.128
Theta	0.264 ± 0.065	0.222 ± 0.114	0.076 ± 0.034
Alpha	0.251 ± 0.080	0.188 ± 0.109	0.085 ± 0.051
Beta	0.074 ± 0.022	0.073 ± 0.064	0.081 ± 0.062
Gamma	0.011 ± 0.012	0.004 ± 0.004	0.004 ± 0.003

Values represent the mean relative spectral power (± SD) within each EEG frequency band.

#### 4.2.2 Results

Relative spectral power differed significantly among seizure states across nearly all conventional EEG frequency bands (Table 5, Figure 3). The largest changes were observed within the theta (*H* = 85.85, *p* < 0.001, *ε*^2^ = 0.570) and delta (*H* = 70.06, *p* < 0.001, *ε*^2^ = 0.463) frequency bands, indicating substantial redistribution of oscillatory power during seizure progression. Significant differences were also identified in the alpha (*H* = 66.50, *p* < 0.001, *ε*^2^ = 0.439) and gamma (*H* = 43.59, *p* < 0.001, *ε*^2^ = 0.283) bands, whereas beta-band power demonstrated only a comparatively small effect (*H* = 6.20, *p* = 0.045, *ε*^2^ = 0.029).

**Table 5:** Overall statistical comparisons for relative spectral power.

Frequency band	Kruskal–Wallis *H*	*p*	*ε* ^2^
Delta	70.06	<0.001	0.463
Theta	85.85	<0.001	0.570
Alpha	66.50	<0.001	0.439
Beta	6.20	0.045	0.029
Gamma	43.59	<0.001	0.283

*ε*^2^ represents the effect size for the Kruskal–Wallis test. Values closer to 1 indicate larger differences among seizure states.

**Figure 3:**
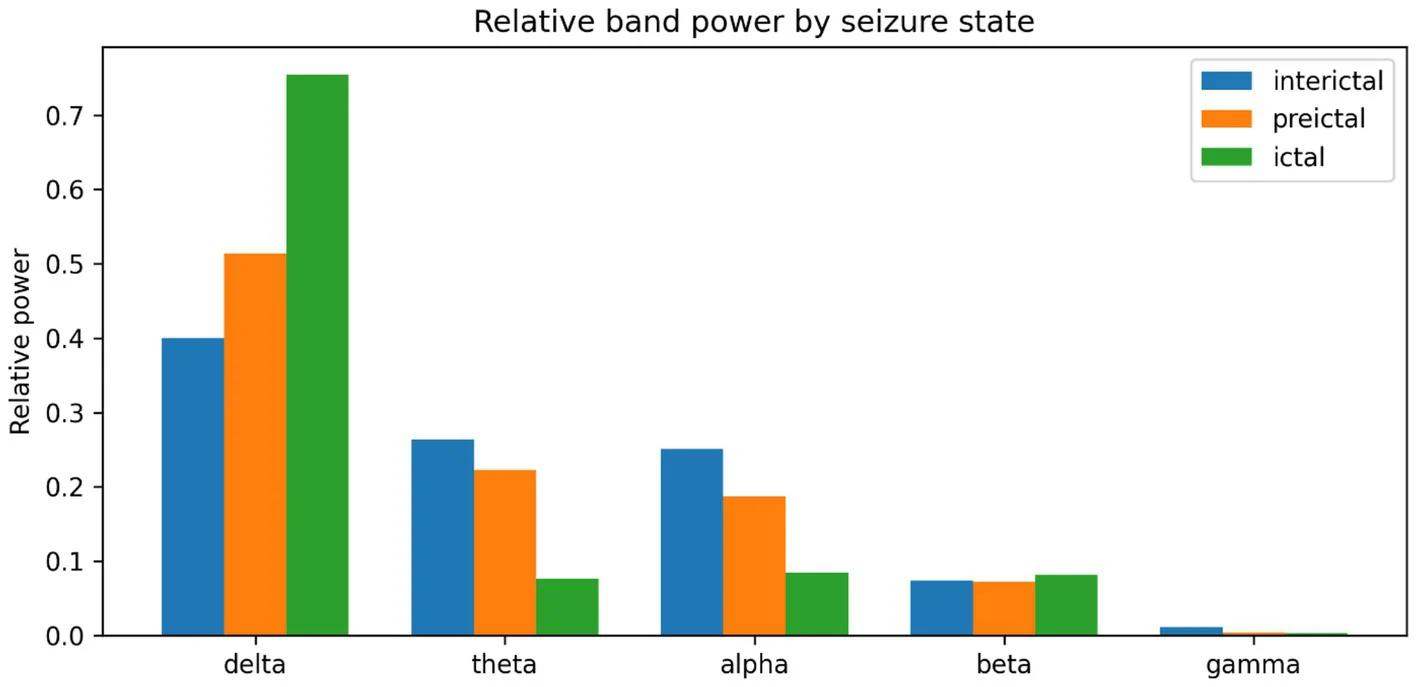
Relative spectral power across seizure states.

Descriptive statistics revealed a progressive increase in delta-band activity from 40.0% of total spectral power during the interictal state to 51.4% during the preictal state and 75.4% during ictal recordings. In contrast, theta-band power declined from 26.4% during interictal recordings to 22.2% during the preictal state and 7.6% during ictal recordings. Alpha-band activity exhibited a similar reduction, decreasing from 25.1 to 18.8 and 8.5%, respectively. Gamma-band activity also declined substantially across seizure states, whereas beta-band power remained comparatively stable. Collectively, these findings indicate that seizure progression was accompanied by a marked redistribution of oscillatory power toward lower-frequency activity, with ictal recordings exhibiting increasing dominance of delta oscillations together with reduced contributions from theta, alpha, and gamma band activity.

The frequency and time domain analyses demonstrated robust increases in signal amplitudes together with a progressive redistribution of spectral power toward lower frequencies during seizure evolution. These observations establish the electrophysiological context for the complexity and dynamical analyses presented in the following sections.

### 4.3 Entropy results

To further characterize changes in the complexity of neural activity across seizure states, three complementary entropy measures were computed for each EEG recording: spectral entropy, permutation entropy, and sample entropy. Together, these metrics quantify different aspects of signal complexity and temporal organization. Spectral entropy reflects the distribution of power across frequencies, permutation entropy quantifies the diversity of temporal patterns within the signal, and sample entropy measures the irregularity of the EEG time series (see Tables 6–8, Figure 4).

**Table 6:** Descriptive statistics for entropy measures.

Measure	Interictal (mean ± SD)	95% CI	Preictal (mean ± SD)	95% CI	Ictal (mean ± SD)	95% CI
Spectral entropy	0.605 ± 0.025	0.599–0.613	0.554 ± 0.062	0.536–0.571	0.466 ± 0.088	0.443–0.491
Permutation entropy	0.772 ± 0.060	0.755–0.789	0.686 ± 0.059	0.670–0.702	0.710 ± 0.063	0.693–0.728
Sample entropy	0.746 ± 0.135	0.711–0.785	0.562 ± 0.130	0.526–0.596	0.456 ± 0.077	0.434–0.476

Values are reported as mean ± standard deviation. CI, 95% bootstrap confidence interval.

**Table 7:** Overall statistical comparisons for entropy measures.

Measure	Kruskal–Wallis *H*	*p*	*ε* ^2^
Spectral entropy	72.49	<0.001	0.480
Permutation entropy	41.35	<0.001	0.268
Sample entropy	88.16	<0.001	0.586

*ε*^2^ represents the Kruskal–Wallis effect size.

**Table 8:** Pairwise *post hoc* comparisons for entropy measures.

Measure	Comparison	Holm-adjusted *p*	Cohen’s *d*
Spectral entropy	Interictal vs. preictal	<0.001	1.09
	Interictal vs. ictal	<0.001	2.16
	Preictal vs. ictal	<0.001	1.15
Permutation entropy	Interictal vs. preictal	<0.001	1.45
	Interictal vs. ictal	<0.001	1.01
	Preictal vs. ictal	0.030	0.39
Sample entropy	Interictal vs. preictal	<0.001	1.39
	Interictal vs. ictal	<0.001	2.65
	Preictal vs. ictal	<0.001	1.00

Holm-adjusted *p*-values are reported to account for multiple pairwise comparisons. Cohen’s *d* provides a standardized estimate of the magnitude of differences between seizure states.

**Figure 4:**
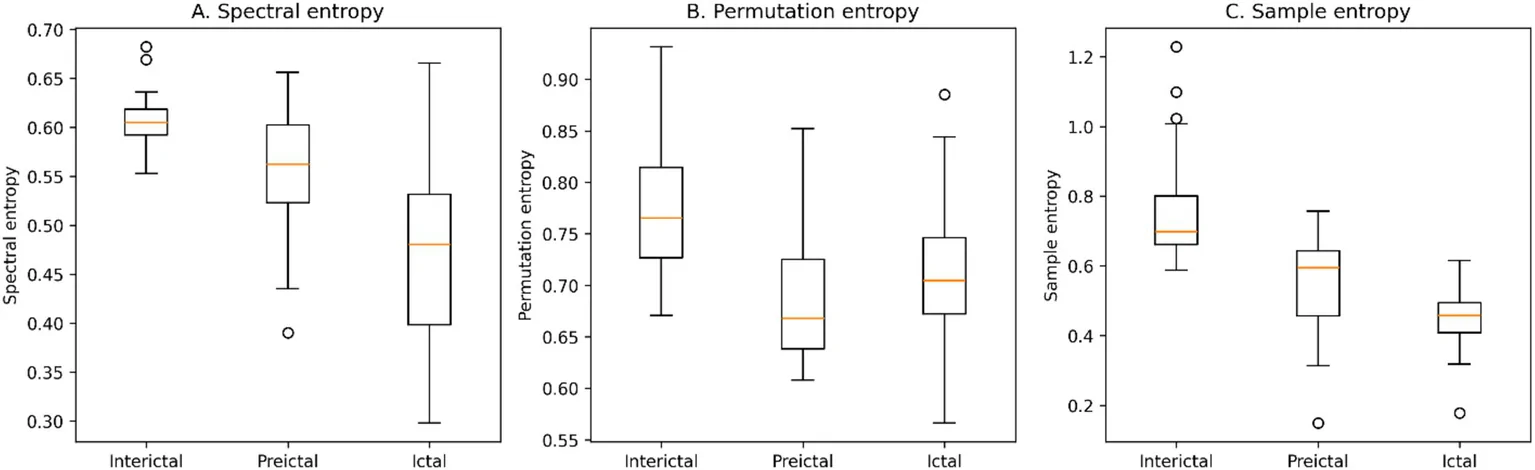
Entropy measures across seizure states. Distribution of spectral entropy, permutation entropy, and sample entropy across interictal, preictal, and ictal EEG recordings. Boxes represent the interquartile range, horizontal lines indicate the median, whiskers denote the range excluding outliers, and individual points represent individual EEG recordings.

#### 4.3.1 Results

Significant differences were observed for all entropy measures across seizure states (Table 7). Spectral entropy decreased progressively from the interictal state (0.605 ± 0.025) to the preictal state (0.554 ± 0.062) and reached its lowest value during ictal recordings (0.466 ± 0.088), corresponding to a large overall effect (*H* = 72.49, *p* < 0.001, *ε*^2^ = 0.480).

Sample entropy exhibited an even larger effect (*H* = 88.16, *p* < 0.001, *ε*^2^ = 0.586). Mean sample entropy declined from 0.746 ± 0.135 during interictal recordings to 0.562 ± 0.130 during the preictal state and 0.456 ± 0.077 during ictal recordings, indicating progressively reduced signal irregularity during seizure evolution.

Permutation entropy also differed significantly among seizure states (*H* = 41.35, *p* < 0.001, *ε*^2^ = 0.268). Mean permutation entropy was greatest during interictal recordings (0.772 ± 0.060), decreased during the preictal state (0.686 ± 0.059), and remained lower during ictal recordings (0.710 ± 0.063) relative to the interictal condition.

Collectively, all three entropy measures demonstrated significant reductions in signal complexity during seizure progression, although the magnitude of these changes differed among entropy metrics.

### 4.4 Phase organization and cross-frequency coupling

To further characterize changes in the temporal organization of neural activity, alpha-band temporal phase concentration and theta/gamma phase amplitude coupling (PAC) were quantified for each EEG recording. Alpha-band temporal phase concentration provides a measure of the consistency of instantaneous phase within the alpha frequency band over time, whereas theta/gamma PAC quantifies the extent to which the amplitude of gamma oscillations is modulated by the phase of slower theta oscillations. Together, these analyses evaluate complementary aspects of temporal organization that are not captured by conventional amplitude or entropy measures (see Tables 9–11, Figures 5, 6).

**Table 9:** Descriptive statistics for phase organization and cross-frequency coupling.

Measure	Interictal (mean ± SD)	95% CI	Preictal (mean ± SD)	95% CI	Ictal (mean ± SD)	95% CI
Alpha-band temporal phase concentration	0.0116 ± 0.0067	0.0098–0.0135	0.0100 ± 0.0042	0.0088–0.0112	0.0191 ± 0.0116	0.0159–0.0225
Theta–gamma PAC (MI)	0.00165 ± 0.00075	0.00146–0.00187	0.00561 ± 0.00891	0.00344–0.00829	0.00444 ± 0.00351	0.00355–0.00548

Values are reported as mean ± standard deviation. CI, 95% bootstrap confidence interval. PAC, phase-amplitude coupling; MI, modulation index.

**Table 10:** Overall statistical comparisons for phase organization and cross-frequency coupling.

Measure	Kruskal–Wallis *H*	*p*	*ε* ^2^
Alpha-band temporal phase concentration	22.49	<0.001	0.139
Theta–gamma PAC	29.25	<0.001	0.185

*ε*^2^ represents the Kruskal–Wallis effect size.

**Table 11:** Pairwise *post hoc* comparisons for phase organization and cross-frequency coupling.

Measure	Comparison	Holm-adjusted *p*	Cohen’s *d*
Alpha-band temporal phase concentration	Interictal vs. preictal	0.406	0.29
	Interictal vs. ictal	<0.001	0.79
	Preictal vs. ictal	<0.001	1.04
Theta–gamma PAC	Interictal vs. preictal	0.224	0.63
	Interictal vs. Ictal	<0.001	1.10
	Preictal vs. Ictal	0.009	0.17

Holm-adjusted *p*-values were used to control for multiple comparisons. Cohen’s *d* values represent standardized effect sizes.

**Figure 5:**
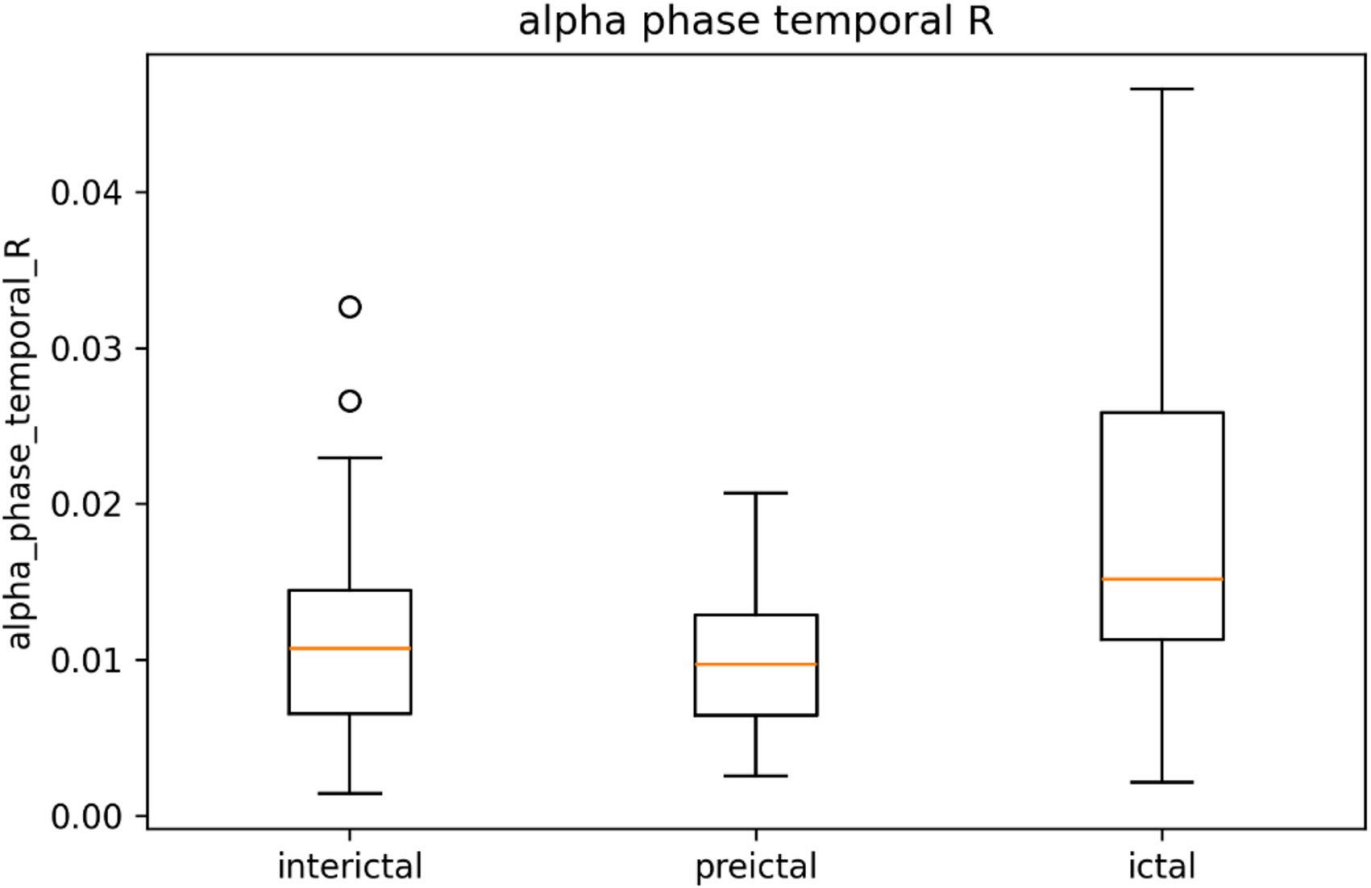
Alpha-band temporal phase concentration across seizure states.

**Figure 6:**
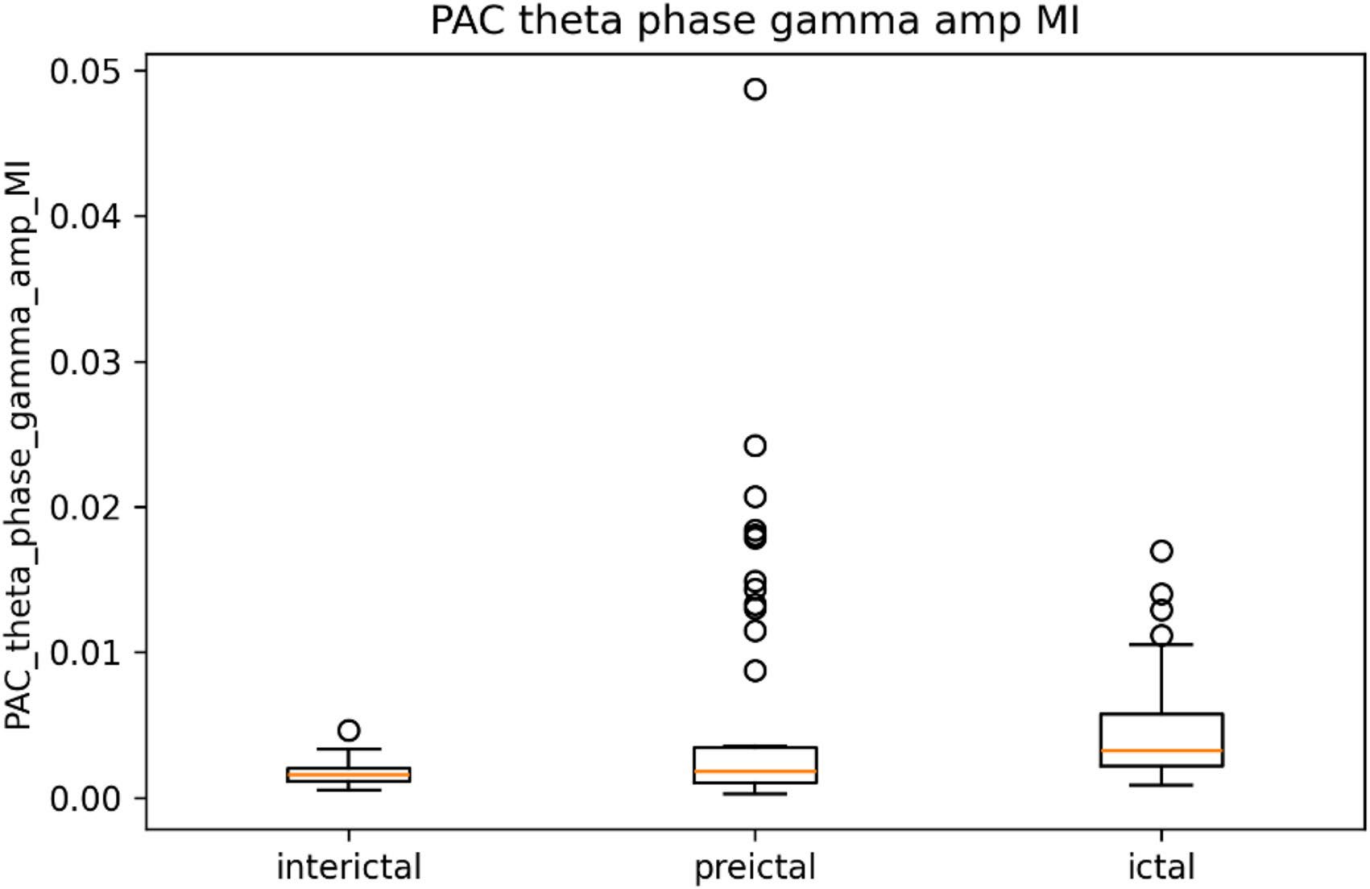
Theta–gamma phase-amplitude coupling across seizure states.

#### 4.4.1 Results

Significant differences were observed among seizure states for both alpha-band temporal phase concentration and theta–gamma phase-amplitude coupling (Tables 10, 11; Figures 5, 6). Alpha-band temporal phase concentration differed significantly across seizure states (*H* = 22.49, *p* < 0.001, *ε*^2^ = 0.139). Mean temporal phase concentration was 0.0116 ± 0.0067 during interictal recordings, 0.0100 ± 0.0042 during preictal recordings, and 0.0191 ± 0.0116 during ictal recordings. *Post hoc* analyses indicated that interictal and preictal recordings did not differ significantly following Holm correction (*p* = 0.406), whereas ictal recordings exhibited significantly greater temporal phase concentration than both interictal (*p* < 0.001, *d* = 0.79) and preictal (*p* < 0.001, *d* = 1.04) recordings.

Theta–gamma phase-amplitude coupling also differed significantly among seizure states (*H* = 29.25, *p* < 0.001, *ε*^2^ = 0.185). Mean modulation index increased from 0.00165 ± 0.00075 during interictal recordings to 0.00561 ± 0.00891 during the preictal state and measured 0.00444 ± 0.00351 during ictal recordings. Pairwise comparisons demonstrated significantly greater PAC during ictal recordings than during the interictal state (*p* < 0.001, *d* = 1.10). Although the overall Kruskal–Wallis test was significant, the comparison between interictal and preictal recordings did not remain significant after Holm correction (*p* = 0.224), while the comparison between preictal and ictal recordings remained significant (*p* = 0.009), despite a small effect size (*d* = 0.17).

Overall, the phase-based analyses identified significant alterations in temporal organization across seizure states. Alpha-band temporal phase concentration demonstrated its largest increase during ictal recordings, whereas theta–gamma phase-amplitude coupling differed primarily between interictal and seizure-associated recordings.

Measures of temporal phase organization and cross-frequency coupling differed significantly among seizure states, although the observed effects were smaller than those identified for amplitude and entropy measures. Alpha-band temporal phase concentration increased during ictal recordings, while theta–gamma phase-amplitude coupling demonstrated significant differences between interictal and seizure-associated activity. These findings provide an additional characterization of seizure-related changes in the temporal organization of EEG dynamics and complement the preceding analyses of signal amplitude and complexity.

### 4.5 Recurrence analysis

To further characterize changes in the temporal organization of EEG dynamics, recurrence quantification analysis (RQA) was performed on the reconstructed state-space trajectories described in Chapter 3. Unlike conventional amplitude or frequency based measures, recurrence analysis quantifies the extent to which neural activity revisits similar dynamical states over time. Four complementary recurrence metrics were evaluated: recurrence rate (RR), determinism (DET), mean diagonal line length (L), and mean state-space displacement (see Tables 12–14, Figures 7–10).

**Table 12:** Descriptive statistics for recurrence analysis.

Measure	Interictal (mean ± SD)	95% CI	Preictal (mean ± SD)	95% CI	Ictal (mean ± SD)	95% CI
Recurrence rate	0.10015 ± 0.00021	0.10010–0.10022	0.10016 ± 0.00023	0.10010–0.10023	0.10001 ± 0.00001	0.10000–0.10001
Determinism	0.908 ± 0.042	0.895–0.919	0.945 ± 0.033	0.936–0.954	0.947 ± 0.026	0.940–0.954
Mean diagonal line length	4.05 ± 0.41	3.94–4.16	5.25 ± 1.38	4.88–5.65	7.70 ± 2.23	7.12–8.36
Mean state-space displacement	0.422 ± 0.061	0.406–0.440	0.334 ± 0.083	0.310–0.356	0.291 ± 0.055	0.277–0.307

Values are reported as mean ± standard deviation. CI, 95% bootstrap confidence interval.

**Table 13:** Overall statistical comparisons for recurrence measures.

Measure	*H*	*p*	*ε* ^2^
Recurrence rate	76.49	<0.001	0.507
Determinism	38.09	<0.001	0.246
Mean diagonal line length	90.55	<0.001	0.602
Mean state-space displacement	65.23	<0.001	0.430

**Table 14:** Pairwise *post hoc* comparisons for recurrence measures.

Measure	Comparison	Holm-adjusted *p*	Cohen’s *d*
Recurrence rate	Interictal vs. preictal	0.997	0.01
	Interictal vs. ictal	<0.001	0.99
	Preictal vs. ictal	<0.001	0.91
Determinism	Interictal vs. preictal	<0.001	0.98
	Interictal vs. ictal	<0.001	1.13
	Preictal vs. ictal	0.997	0.09
Mean diagonal line length	Interictal vs. preictal	<0.001	1.17
	Interictal vs. ictal	<0.001	2.27
	Preictal vs. ictal	<0.001	1.32
Mean state-space displacement	Interictal vs. preictal	<0.001	1.22
	Interictal vs. ictal	<0.001	2.25
	Preictal vs. ictal	0.003	0.60

Holm-adjusted *p*-values were used to control for multiple pairwise comparisons. Cohen’s *d* values represent standardized effect sizes.

**Figure 7:**
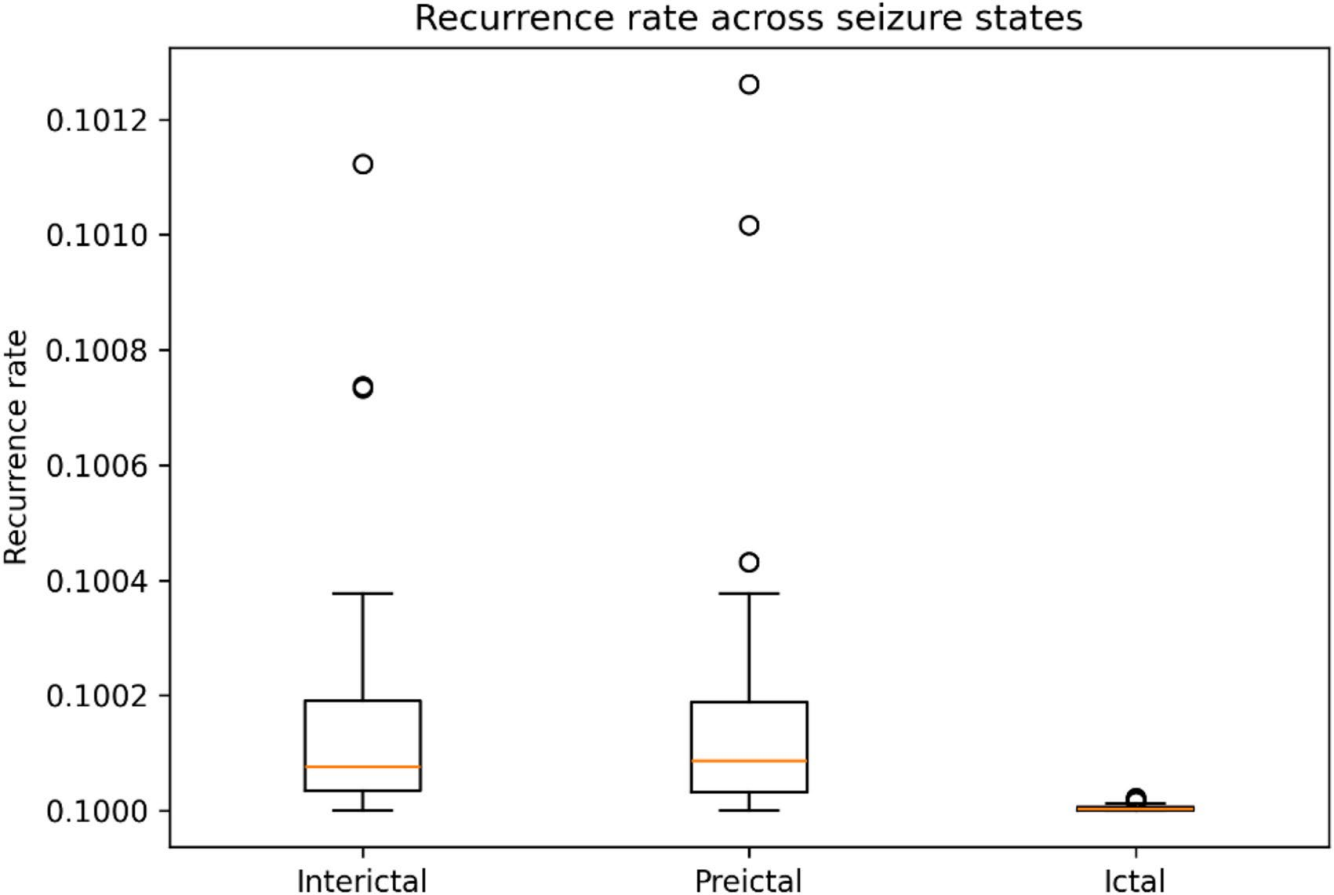
Recurrence rate across seizure states.

**Figure 8:**
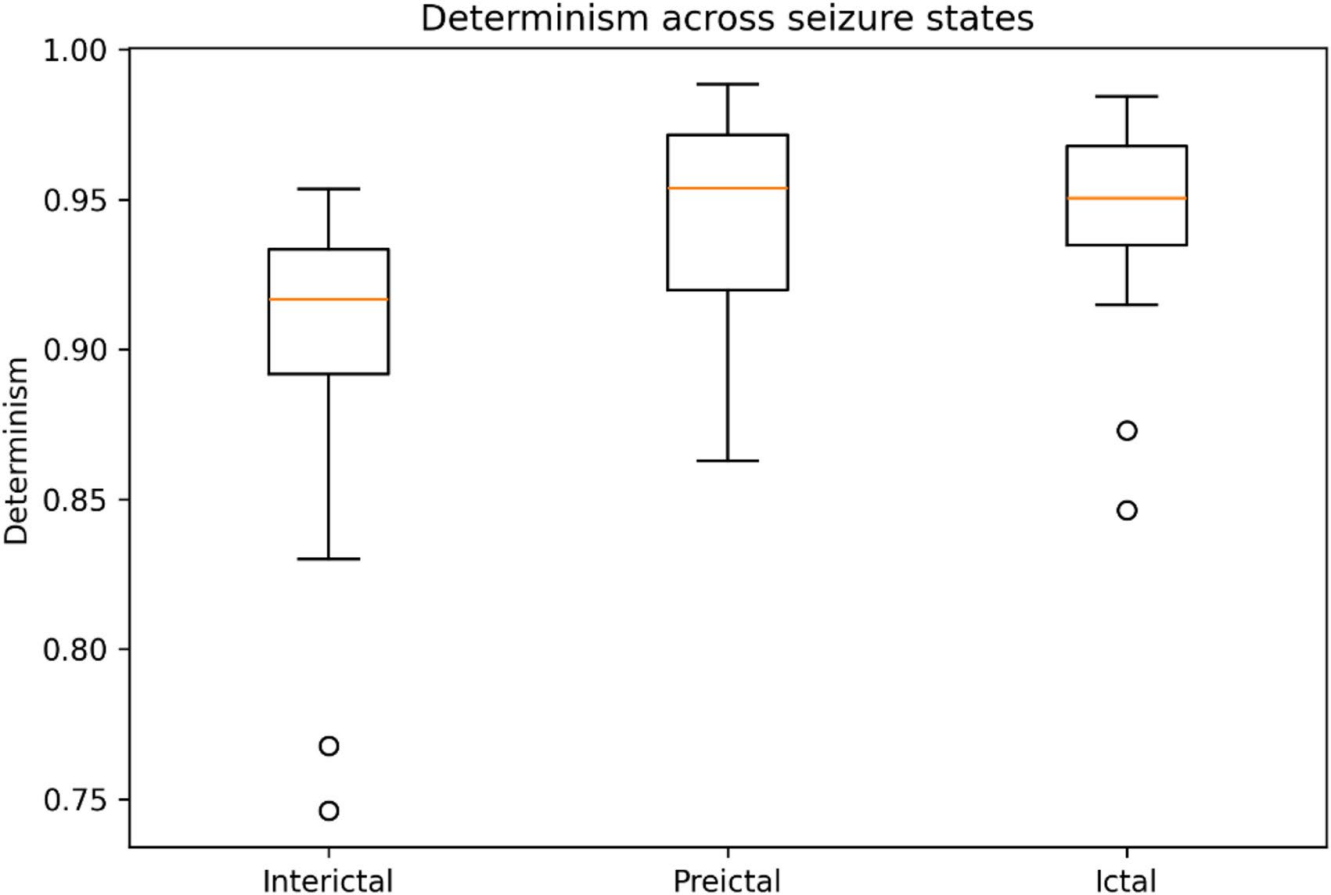
Determinism across seizure states.

**Figure 9:**
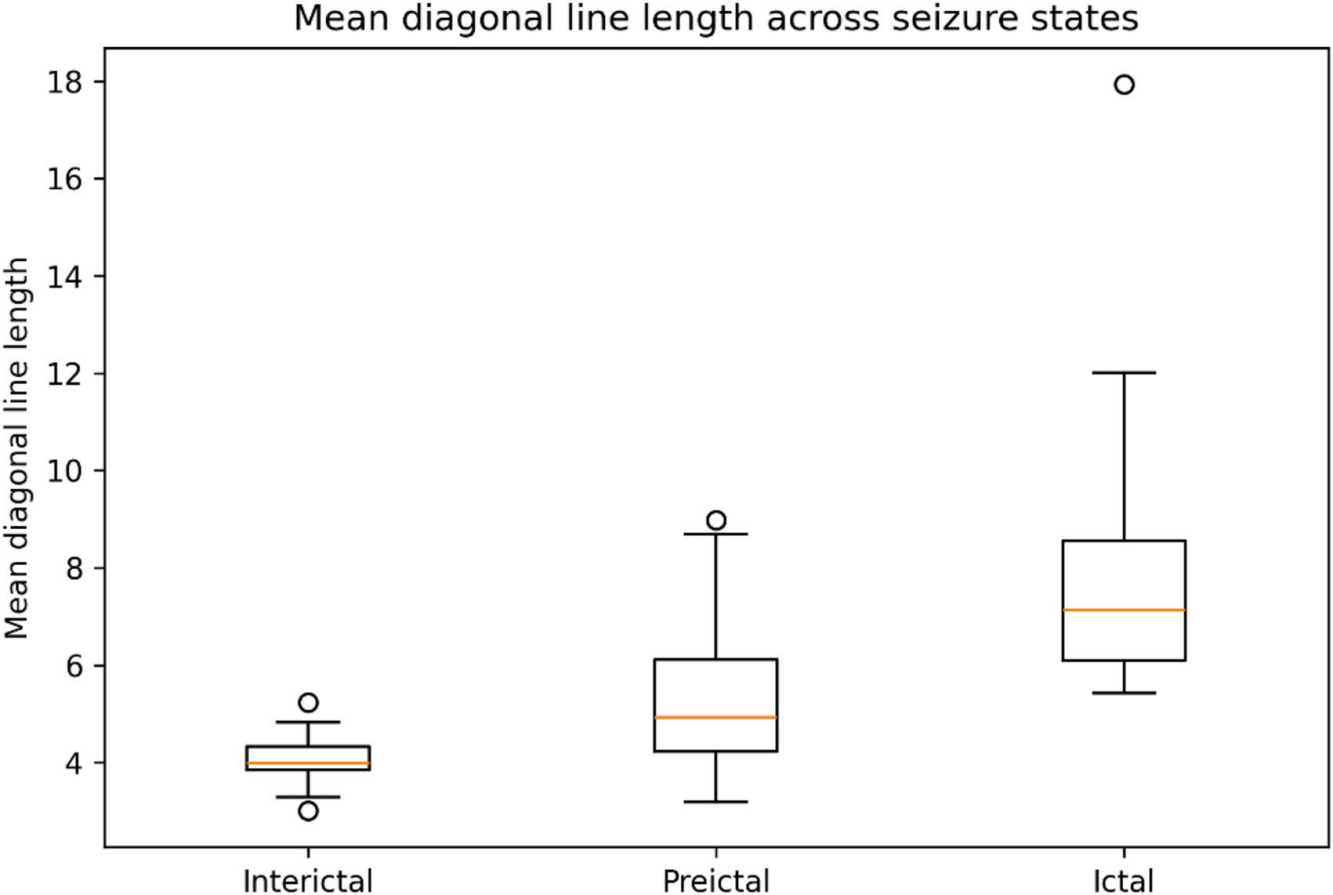
Mean diagonal line length across seizure states.

**Figure 10:**
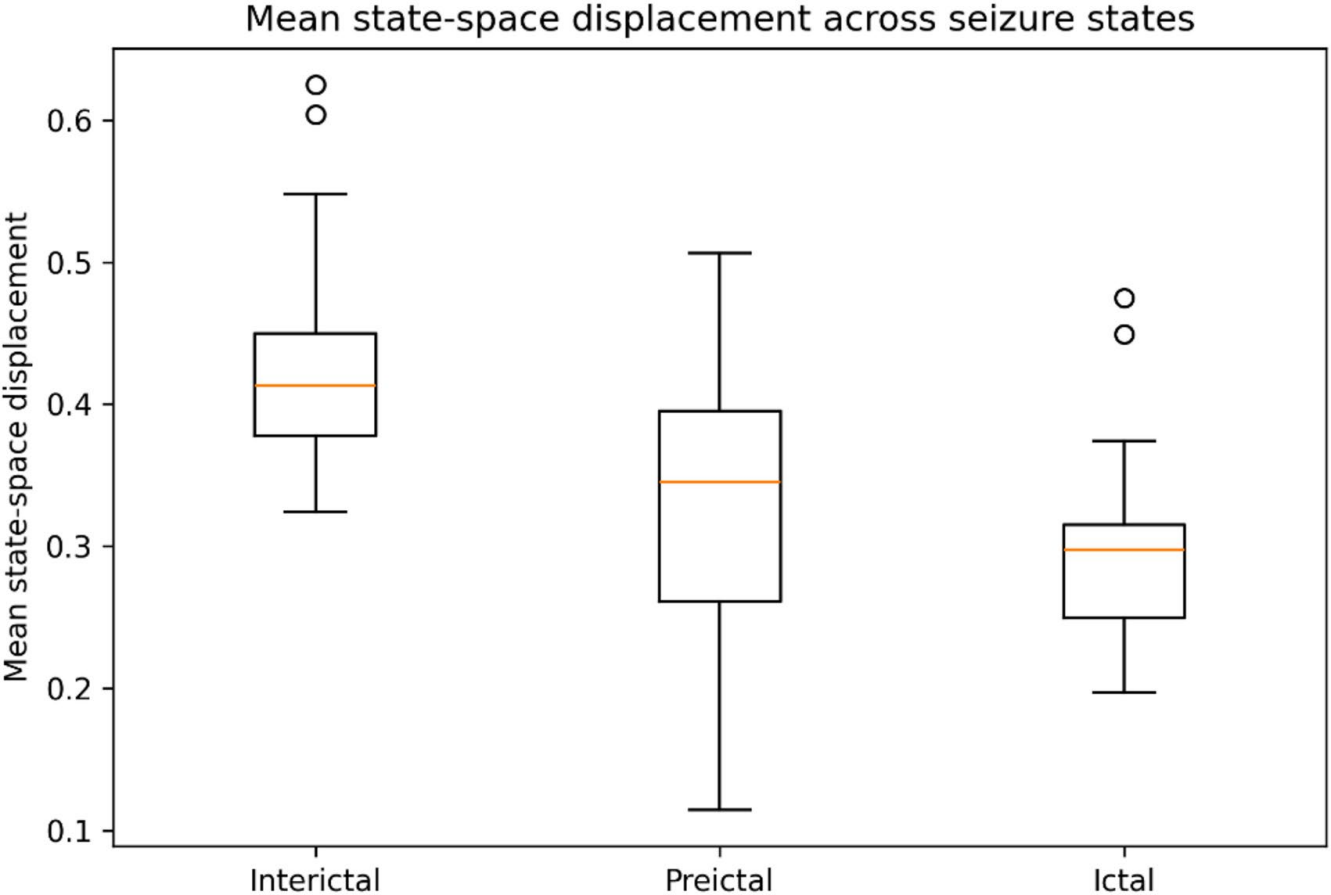
Mean state-space displacement across seizure states.

#### 4.5.1 Results

Significant differences were observed among seizure states for all recurrence measures (Tables 13, 14; Figures 7–10). Recurrence rate differed significantly across seizure states (*H* = 76.49, *p* < 0.001, *ε*^2^ = 0.507). Although the numerical differences in recurrence rate were small because the recurrence threshold was normalized to produce a target recurrence density, *post hoc* analyses demonstrated significantly lower recurrence rates during ictal recordings than during both interictal and preictal recordings (both *p* < 0.001). No significant difference was observed between interictal and preictal recordings (*p* = 0.997).

Determinism also differed significantly among seizure states (*H* = 38.09, *p* < 0.001, *ε*^2^ = 0.246). Mean determinism increased from 0.908 ± 0.042 during interictal recordings to 0.945 ± 0.033 during the preictal state and 0.947 ± 0.026 during ictal recordings. Pairwise comparisons indicated significant differences between interictal recordings and both seizure-associated states (both *p* < 0.001), whereas the preictal and ictal groups did not differ significantly (*p* = 0.997).

Mean diagonal line length exhibited the largest overall effect among the recurrence measures (*H* = 90.55, *p* < 0.001, *ε*^2^ = 0.602). Mean values increased progressively from 4.05 ± 0.41 during interictal recordings to 5.25 ± 1.38 during the preictal state and 7.70 ± 2.23 during ictal recordings. All pairwise comparisons remained significant following Holm correction (all *p* < 0.001), with large effect sizes for each comparison.

Mean state-space displacement also differed significantly across seizure states (*H* = 65.23, *p* < 0.001, *ε*^2^ = 0.430). Mean displacement decreased from 0.422 ± 0.061 during interictal recordings to 0.334 ± 0.083 during the preictal state and 0.291 ± 0.055 during ictal recordings. Pairwise comparisons demonstrated significant differences among all seizure states, with the largest effect observed between the interictal and ictal conditions (*d* = 2.25).

Collectively, the recurrence analyses demonstrated systematic alterations in the temporal organization of reconstructed EEG state-space trajectories across seizure states. The largest effects were observed for mean diagonal line length and recurrence rate, while determinism and mean state-space displacement also differed significantly among seizure conditions. Recurrence analysis identified significant changes in multiple measures of EEG state-space dynamics during seizure progression. Mean diagonal line length demonstrated the largest effect, followed by recurrence rate and mean state-space displacement. Determinism increased during seizure-associated recordings relative to the interictal state. Together, these findings provide a quantitative description of changes in the temporal organization of reconstructed EEG dynamics and complement the amplitude, spectral, entropy, and phase-based analyses presented in the preceding sections.

### 4.6 Integrated electrophysiological findings

The analyses presented in Sections 4.2–4.5 collectively demonstrated consistent and statistically significant differences among interictal, preictal, and ictal EEG recordings across multiple complementary measures of neural activity. Although each analytical approach quantified a distinct characteristic of the EEG signal, the results revealed a coherent pattern of changes accompanying seizure progression. The largest effects were observed for measures of overall signal amplitude. Both RMS amplitude and the Hilbert envelope demonstrated substantial increases during ictal recordings relative to the interictal and preictal states, with very large effect sizes (*ε*^2^ ≈ 0.66). These findings indicate that seizure activity was accompanied by marked increases in the magnitude of the recorded electrical signal.

Frequency-domain analyses demonstrated systematic redistribution of oscillatory activity across seizure states. Relative delta-band power increased progressively from the interictal to the ictal state, whereas theta-, alpha-, and gamma-band contributions generally decreased. Changes in dominant oscillatory frequency were statistically significant but comparatively small, suggesting that alterations in spectral composition were driven primarily by redistribution of power among frequency bands rather than large shifts in the peak oscillatory frequency.

Measures of signal complexity also differed substantially among seizure states. Spectral entropy and sample entropy exhibited progressive reductions from the interictal to the ictal condition, indicating lower signal complexity during seizure activity. Permutation entropy followed a distinct pattern characterized by a reduction during the preictal state followed by a modest increase during ictal recordings, although values remained lower than those observed during the interictal condition. Collectively, these findings demonstrate that different entropy measures captured complementary aspects of EEG organization.

Analyses of temporal organization likewise identified significant differences across seizure states. Alpha-band temporal phase concentration increased during ictal recordings, while theta–gamma phase-amplitude coupling differed significantly among seizure states, primarily distinguishing interictal recordings from seizure-associated activity. These findings indicate measurable alterations in temporal organization beyond those captured by amplitude or spectral analyses alone.

Recurrence quantification analysis further demonstrated significant changes in reconstructed EEG dynamics. Mean diagonal line length exhibited the largest effect among the recurrence measures, followed by recurrence rate and mean state-space displacement. Determinism increased during seizure-associated recordings relative to the interictal state, while mean state-space displacement decreased progressively across seizure states. Together, these findings indicate systematic differences in the temporal structure of reconstructed EEG trajectories.

Across all analyses, the largest effects were consistently observed for measures of signal amplitude, entropy, and recurrence dynamics. Although the magnitude of individual effects varied among analytical methods, every primary category of analysis identified statistically significant differences among seizure states. This convergence across independent quantitative measures indicates that seizure progression was associated with widespread changes in multiple electrophysiological properties of the EEG signal. The results presented in this chapter establish that large-scale neural dynamics differ systematically across seizure states. Whether these measurable changes correspond to broader principles governing the emergence of unified thought is a theoretical question considered in the Discussion.

Horizontal bar chart illustrating the magnitude of the Kruskal–Wallis effect size (ε^2^) for each primary electrophysiological measure evaluated in the study. Measures are ordered from the largest to the smallest effect size. Signal amplitude (RMS amplitude and Hilbert envelope), sample entropy, and recurrence metrics exhibited the largest effects, whereas dominant frequency and beta-band relative power demonstrated comparatively small effects (see Table 15, Figure 11).

**Table 15:** Integrated summary of primary electrophysiological findings.

Domain	Principal measure	Largest effect (*ε*^2^)	Primary observation
Signal amplitude	RMS amplitude	0.658	Large increase in overall EEG signal magnitude during seizures
Instantaneous amplitude	Hilbert envelope	0.658	Increased signal envelope during seizure activity
Spectral organization	Theta relative power	0.570	Redistribution of oscillatory power across frequency bands
Signal complexity	Sample entropy	0.586	Progressive reduction in signal complexity
Temporal phase organization	Alpha temporal phase concentration	0.139	Increased temporal phase concentration during ictal recordings
Cross-frequency coupling	Theta–gamma PAC	0.185	Altered theta–gamma coupling across seizure states
Recurrence dynamics	Mean diagonal line length	0.602	Increased persistence of recurrent state-space trajectories
State-space geometry	Mean state-space displacement	0.430	Reduced movement through reconstructed state space

The table summarizes the principal findings for each analytical domain. Values represent the largest effect size (*ε*^2^) observed within each category and are intended to provide an overview of the electrophysiological changes identified across the study.

**Figure 11:**
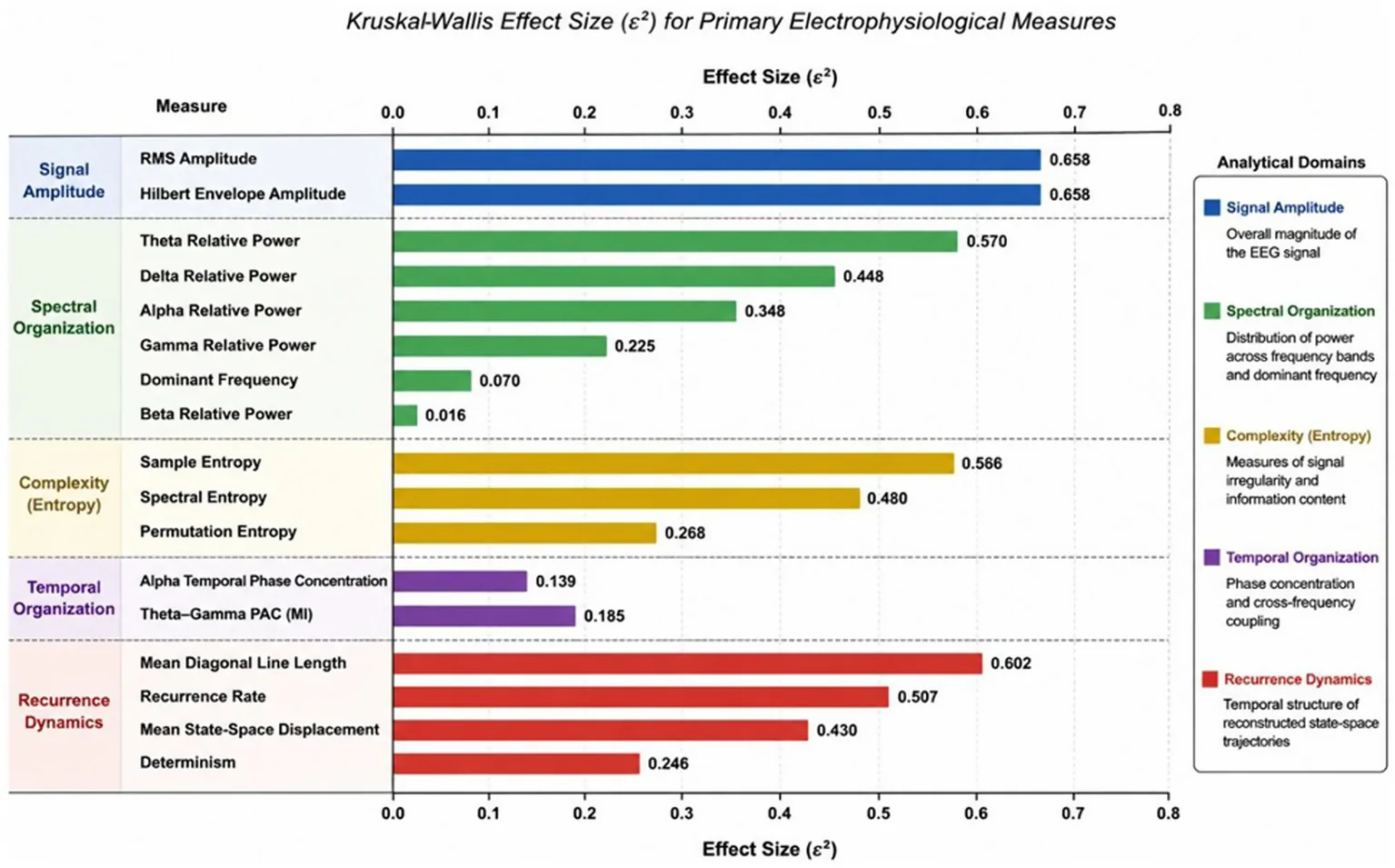
Summary of effect sizes across analytical domains.

## 5 Chapter 5: Discussion

### 5.1 Principal findings

The present study investigated whether established electrophysiological measures obtained from EEG recordings could be interpreted collectively within a mathematical framework describing evolving neural dynamics. Analyses of 150 interictal, preictal, and ictal EEG recordings demonstrated coordinated changes across multiple complementary electrophysiological observables, including signal amplitude, spectral organization, entropy, temporal phase dynamics, cross-frequency coupling, and recurrence structure. Although each analysis quantified a different property of the EEG signal, the results consistently indicated that seizure progression is associated with systematic changes across multiple dimensions of neural activity rather than isolated alterations in a single electrophysiological feature.

The largest effects were observed for measures of signal amplitude. Both RMS amplitude and the Hilbert envelope increased substantially during ictal recordings, indicating marked increases in the magnitude of recorded electrical activity. Spectral analyses further demonstrated a redistribution of oscillatory power toward lower-frequency activity, characterized by increasing delta-band dominance together with reductions in theta-, alpha-, and gamma-band contributions. By comparison, dominant oscillatory frequency exhibited relatively small changes, suggesting that seizure evolution was characterized primarily by redistribution of spectral energy rather than the emergence of entirely new oscillatory rhythms. Measures of signal complexity likewise changed systematically across seizure states. Spectral entropy and sample entropy progressively decreased during seizure progression, whereas permutation entropy followed a distinct temporal trajectory characterized by an initial reduction during the preictal period followed by a modest increase during ictal activity. These findings indicate that different entropy measures capture complementary aspects of neural dynamics and emphasize that no single complexity metric provides a complete description of seizure-related changes in EEG organization.

Additional analyses of temporal organization and nonlinear dynamics revealed widespread changes beyond amplitude and spectral content alone. Alpha-band temporal phase concentration, theta-gamma phase-amplitude coupling, and recurrence quantification measures all differed significantly among seizure states. In particular, recurrence analyses demonstrated large effects for recurrence determinism and mean diagonal line length, indicating systematic changes in the temporal organization of reconstructed EEG trajectories during seizure progression. Taken together, these findings demonstrate that seizure evolution is characterized by coordinated alterations across multiple complementary electrophysiological domains. Importantly, the present study does not interpret these observations as direct measurements of candidate neural configurations, unified thought, or conscious experience. Rather, they provide quantitative descriptions of evolving neural dynamics that form the empirical foundation for evaluating the mathematical framework proposed in this dissertation. The following sections consider how these established electrophysiological observations relate to existing theories of neural organization and discuss the extent to which wave-function formalism may provide a useful representational framework for interpreting their collective behavior.

### 5.2 Relationship to existing EEG literature

The electrophysiological findings of the present study are broadly consistent with the established epilepsy EEG literature. Previous investigations have demonstrated that seizure activity is associated with increased signal amplitude, redistribution of spectral power toward lower-frequency oscillations, reductions in signal complexity, alterations in cross-frequency interactions, and changes in nonlinear dynamical organization (Schomer and Lopes da Silva, 2018; Fisher et al., 2017; Kramer and Cash, 2012; Guerrero-Aranda et al., 2023). Collectively, these studies indicate that seizure progression is accompanied by coordinated changes across multiple complementary electrophysiological measures rather than isolated alterations in any single property of the EEG signal.

The present findings reproduce these well-established observations. Ictal recordings exhibited marked increases in signal amplitude, progressive redistribution of oscillatory power toward lower-frequency activity, reductions in spectral and sample entropy, alterations in phase organization and cross-frequency coupling, and significant changes in recurrence dynamics. Individually, none of these observations are unexpected. Rather, they are consistent with a substantial body of evidence demonstrating that epileptic seizures are characterized by systematic changes in the temporal organization of neural activity (Jiruska et al., 2013; Schomer and Lopes da Silva, 2018; Marwan et al., 2007). Importantly, the present study does not propose any of these electrophysiological measures as novel biomarkers of seizure activity. Measures such as RMS amplitude, spectral entropy, phase-amplitude coupling, and recurrence quantification analysis have each been extensively investigated within clinical and computational neuroscience. The novelty of the present work therefore does not lie in the development of new EEG analyses, but in the proposal that these established electrophysiological observables may be interpreted collectively within a common mathematical representation of evolving neural dynamics.

This distinction is important because contemporary EEG research increasingly recognizes that no single electrophysiological measure provides a complete description of neural dynamics. Signal amplitude characterizes the magnitude of synchronized neural activity, entropy quantifies aspects of signal complexity and predictability, oscillatory analyses describe spectral organization, phase-amplitude coupling characterizes interactions across temporal scales, and recurrence quantification analysis captures deterministic structure within evolving neural trajectories (Richman and Moorman, 2000; Bandt and Pompe, 2002; Tort et al., 2010; Marwan et al., 2007). Each of these measures therefore provides complementary information regarding the temporal organization of the recorded EEG signal rather than an independent description of neural activity.

The mathematical framework proposed in this dissertation builds upon this observation by treating these complementary electrophysiological measures as different descriptors of a single evolving neural state. Rather than interpreting amplitude, entropy, oscillatory organization, phase dynamics, cross-frequency coupling, and recurrence characteristics independently, the proposed wave-function formalism provides a unified state-space representation within which these observables may be interpreted collectively. Importantly, this framework does not replace existing EEG analyses or claim to identify new physiological mechanisms. Instead, it offers a mathematical language for organizing multiple established electrophysiological observables within a common representational framework. Accordingly, the contribution of the present study is twofold. First, the analyses reproduce and integrate well-established electrophysiological findings reported throughout the epilepsy literature using a comprehensive quantitative analysis pipeline. Second, they provide the empirical basis for evaluating whether multiple complementary EEG observables can be represented within a unified mathematical framework describing evolving neural dynamics. The principal contribution of the present work is therefore not a new electrophysiological measure, but a new mathematical interpretation of established EEG observables. The broader theoretical implications of this representational framework are considered in the following sections.

### 5.3 Entropy as one component of neural coordination

Entropy-based approaches have become increasingly influential in computational neuroscience because they provide quantitative measures of signal complexity, predictability, and information content across a wide range of physiological and pathological brain states. Reductions in EEG entropy have consistently been reported during epileptic seizures, general anesthesia, and disorders of consciousness, where they are commonly interpreted as reflecting increasingly constrained neural dynamics (Bruhn et al., 2000; Richman and Moorman, 2000; Bandt and Pompe, 2002; Mediano et al., 2022). Within several contemporary theories of consciousness, greater neural complexity is likewise proposed to correspond to a richer repertoire of accessible neural states (Rosas et al., 2020; Mediano et al., 2022).

The findings of the present study are broadly consistent with this literature. Spectral entropy and sample entropy both decreased significantly during seizure progression, indicating progressively reduced complexity of large-scale neural activity. These observations agree with numerous previous investigations demonstrating that epileptic seizures are accompanied by increasingly regular and predictable electrophysiological dynamics (Acharya et al., 2015; Bruhn et al., 2000). At the same time, permutation entropy followed a distinct temporal profile, suggesting that different entropy measures capture complementary aspects of neural organization rather than a single underlying property.

Importantly, entropy represented only one component of the broader electrophysiological changes observed in the present study. Significant alterations were also identified in signal amplitude, oscillatory organization, phase dynamics, cross-frequency coupling, and recurrence characteristics. Each of these measures describes a different aspect of large-scale neural organization. Amplitude reflects the magnitude of coordinated neural activity, entropy characterizes complexity and predictability, oscillatory analyses describe spectral organization, phase-amplitude coupling quantifies interactions across temporal scales, and recurrence analysis evaluates deterministic structure within evolving neural trajectories. No single measure therefore provides a complete description of the underlying neural dynamics.

These observations motivate one aspect of the mathematical framework proposed in this dissertation. Rather than interpreting these electrophysiological measures independently, the proposed dynamical neural-field framework treats them as complementary descriptors of a common evolving neural-field landscape. Within this interpretation, entropy is not regarded as a direct measure of unified thought or consciousness, but as one observable property contributing to the broader organization of large-scale neural dynamics. The proposed framework therefore extends entropy-based approaches by emphasizing coordinated changes across multiple electrophysiological observables rather than assigning explanatory priority to any individual metric.

This distinction also clarifies the relationship between the present framework and existing entropy-based models of consciousness. The proposed neural-field framework does not reject the importance of neural complexity or information-rich dynamics. Rather, it suggests that complexity alone may provide an incomplete characterization of large-scale neural organization. Neural systems exhibiting similar entropy values may nevertheless differ substantially in their oscillatory coordination, temporal organization, cross-frequency interactions, and state-space dynamics. Accordingly, the present framework proposes that unified thought is more appropriately understood as emerging from coordinated organization across multiple dimensions of neural dynamics than from entropy alone.

Importantly, the present study does not propose a new measure of neural complexity. Instead, it introduces a conceptual framework in which multiple established electrophysiological observables including entropy, are interpreted collectively as complementary descriptors of an evolving neural-field landscape. This interpretation provides a mechanistic foundation for understanding how distributed neural dynamics may become transiently organized into a unified thought while remaining fully compatible with established electrophysiological measures. The relationship between this proposed mechanism and existing theories of large-scale neural integration is considered in the following section.

### 5.4 Relationship to integrated information theory

Integrated Information Theory (IIT) is one of the most influential contemporary theoretical frameworks for understanding consciousness and has provided one of the most rigorous mathematical approaches to relating neural organization to conscious experience. Since its original formulation and subsequent development (Tononi, 2004, 2008), IIT has undergone substantial mathematical development through IIT 3.0 (Oizumi et al., 2014) and, more recently, IIT 4.0 (Albantakis et al., 2023). Across these formulations, the central proposition remains that conscious experience corresponds to the degree of integrated information generated by a physical system. Rather than emphasizing the amount of neural activity alone, IIT proposes that consciousness depends upon the irreducible causal structure generated by interactions among distributed system elements, quantified by the measure Φ (phi) (Tononi et al., 2016).

The electrophysiological findings reported in the present study are broadly consistent with the general principles motivating IIT. Significant alterations were observed across multiple complementary measures of neural dynamics, including signal amplitude, oscillatory organization, entropy, phase dynamics, cross-frequency coupling, and recurrence characteristics. Collectively, these findings indicate that seizure progression involves coordinated changes in distributed neural organization rather than isolated alterations in individual neural regions. Such observations are compatible with the broader view that large-scale interactions play an essential role in supporting complex brain function (Tononi et al., 2016; Albantakis et al., 2023).

Importantly, the present study does not directly estimate integrated information or calculate Φ. Consequently, these findings should not be interpreted as empirical tests of Integrated Information Theory. Instead, the results demonstrate that large-scale neural organization changes systematically across seizure states and provide quantitative observations that may be interpreted within several contemporary theoretical frameworks, including IIT. The principal distinction between IIT and the present framework lies in the explanatory target. Integrated Information Theory seeks to quantify the degree of information integration within a system and proposes that this quantity corresponds to consciousness. By contrast, the present framework does not introduce a new measure of integration or attempt to replace Φ. Instead, it proposes a dynamical description of how multiple simultaneously active neural configurations evolve within a common neural-field landscape and how one configuration may become transiently dominant to produce the current unified thought.

Accordingly, the present framework should not be viewed as an alternative measure of consciousness, but as a complementary dynamical interpretation of large-scale neural organization. Whereas IIT emphasizes the quantity of integrated information, the proposed neural-field framework emphasizes the temporal evolution of neural coordination. In this interpretation, amplitude, entropy, oscillatory organization, phase dynamics, cross-frequency coupling, and recurrence characteristics are not independent biomarkers but complementary descriptors of the evolving neural-field landscape. The proposed framework therefore addresses a different explanatory question: not how much information is integrated, but how coordinated neural dynamics become organized into the transient neural configuration corresponding to a unified thought.

This distinction is particularly important because systems exhibiting similar degrees of integrated information may nevertheless differ substantially in their temporal organization, oscillatory coordination, and dynamical evolution. The present framework therefore suggests that understanding large-scale neural dynamics requires consideration of both integration and temporal organization. From this perspective, integrated information represents one important aspect of distributed neural activity, while the evolving neural-field landscape provides a complementary mathematical description of how that organization changes through time.

The proposed framework should therefore be viewed as complementary to Integrated Information Theory rather than competitive with it. IIT provides a rigorous account of information integration across distributed neural systems, whereas the present framework proposes a dynamical mechanism through which multiple simultaneously active neural configurations become transiently organized into one unified thought. Whether these complementary perspectives ultimately describe different aspects of the same underlying neural process remains an important question for future investigation.

### 5.5 Relationship to electromagnetic field theories

Electromagnetic field theories of consciousness represent one of the most influential attempts to explain how distributed neural activity gives rise to unified conscious experience (Jirsa and Haken, 1996). Rather than locating consciousness within individual neurons or isolated cortical regions, these theories propose that large-scale electromagnetic fields generated by synchronized neural activity provide the substrate through which distributed information becomes integrated across the brain. Among the most extensively developed formulations are Johnjoe McFadden’s Conscious Electromagnetic Information (CEMI) theory (McFadden, 2002, 2020, 2023) and Susan Pockett’s electromagnetic field theory of consciousness (Pockett, 2000, 2012). Although these frameworks differ in important theoretical details, both assign a fundamental role to large-scale electromagnetic field organization in the generation of conscious experience.

Several observations reported in the present study are broadly compatible with the motivations underlying these theories. Seizure progression was associated with coordinated alterations in signal amplitude, oscillatory organization, entropy, phase dynamics, cross-frequency coupling, and recurrence characteristics, indicating that epileptic seizures involve widespread reorganization of large-scale neural activity rather than isolated local neuronal events. In this respect, both electromagnetic field theories and the present framework emphasize that understanding brain function requires consideration of distributed neural dynamics rather than localized neural activity alone.

Despite these similarities, an important conceptual distinction separates the present framework from existing electromagnetic field theories. Neither the empirical analyses nor the theoretical framework developed in this dissertation propose that the brain’s electromagnetic field constitutes consciousness or serves as its physical substrate. Instead, the neural field introduced in the present work is a mathematical representation of measurable large-scale neural dynamics inferred from electrophysiological observations. The analytic signal,

$$\Psi (t)=A(t)e^{\mathrm{i\phi}(t)}$$

provides a compact mathematical description of evolving neural activity by combining instantaneous amplitude and phase within a single formalism. The neural field therefore functions as a descriptive representation of neural dynamics rather than as a claim regarding the ontological basis of consciousness.

A second distinction concerns causality. CEMI theory proposes that the brain’s electromagnetic field plays an active causal role by influencing neuronal firing through reciprocal interactions between neural tissue and the surrounding electromagnetic field (McFadden, 2023). Pockett likewise argues that particular spatiotemporal electromagnetic field configurations are themselves conscious experiences (Pockett, 2012). The present framework remains intentionally agnostic regarding these causal and ontological questions. Rather than proposing that the neural field possesses independent causal properties, the framework focuses on describing the measurable organization and temporal evolution of large-scale neural dynamics using established electrophysiological observables. Consequently, the proposed model should be understood as a dynamical description of neural organization rather than a theory regarding the physical substrate of consciousness.

The principal contribution of the present framework lies in the mechanism it proposes for the emergence of unified thought. Building upon the mathematical framework introduced in Chapter 2, the evolving neural field is conceptualized as a landscape containing multiple simultaneously active neural configurations. Unified thought is proposed to emerge when one configuration becomes transiently dominant through coordinated neural dynamics while alternative configurations remain dynamically active outside immediate awareness. This mechanism differs fundamentally from electromagnetic field theories by focusing on the temporal organization of measurable neural activity rather than on the causal properties of the electromagnetic field itself.

Accordingly, the proposed framework should not be viewed as an alternative measure of consciousness or a replacement for integrated information. Rather, it is intended as a complementary mathematical framework for representing evolving neural dynamics. Whereas IIT emphasizes the quantity of information integration, the present framework emphasizes the coordinated evolution of multiple electrophysiological observables within a common state-space representation. Signal amplitude, entropy, oscillatory organization, phase dynamics, cross-frequency coupling, and recurrence structure are therefore interpreted not as independent biomarkers but as complementary mathematical descriptors of the evolving neural state.

The proposed framework should therefore be viewed as complementary to Integrated Information Theory rather than competitive with it. IIT provides a rigorous mathematical description of information integration across interacting system elements. The framework proposed in this dissertation instead investigates whether wave-function formalism provides a useful mathematical language for representing the continuous evolution of neural dynamics measured using EEG. These approaches therefore address different aspects of neural organization: IIT focuses on quantifying integration, whereas the present framework focuses on representing the temporal evolution of multiple complementary electrophysiological observables within a common mathematical state space. Whether these two perspectives ultimately describe different mathematical properties of the same underlying neural processes remains an important question for future investigation.

### 5.6 Wave-function formalism as a mathematical representation of neural dynamics

The empirical findings presented in this study demonstrate that seizure progression is associated with coordinated changes across multiple complementary electrophysiological observables, including signal amplitude, oscillatory organization, entropy, phase dynamics, cross-frequency coupling, and recurrence structure. These observations are consistent with a substantial body of epilepsy EEG literature demonstrating that neural activity evolves through systematic changes across multiple dimensions of electrophysiological organization rather than through isolated alterations in any single signal property (Jiruska et al., 2013; Schomer and Lopes da Silva, 2018; Kramer and Cash, 2012). The present work builds upon these established observations by asking whether these complementary electrophysiological measures can be represented within a common mathematical formalism describing the evolution of neural dynamics through time.

At the empirical level, the neural signal is represented using the analytic signal obtained from the Hilbert transform,

$$\Psi (t)=A(t)e^{\mathrm{i\phi}(t)}$$

where *A* (*t*) represents the instantaneous signal amplitude and ϕ(t) represents the instantaneous phase. This complex-valued representation is a standard mathematical tool in signal processing and electrophysiology that provides a compact description of oscillatory neural activity (Le Van Quyen et al., 2001; Cohen, 2014). Within the present framework, this expression should not be interpreted as a quantum-mechanical wave function or as a representation of the brain’s physical electromagnetic field. Rather, it serves as a mathematical representation that simultaneously captures complementary measurable properties of evolving EEG dynamics.

Building upon this measurable representation, the proposed framework conceptualizes the evolving neural state as the superposition of multiple simultaneously active neural configurations,

Ψ(t)=i=1∑Nci(t)Ψi(t)

where Ψ*i* (*t*) represents a candidate neural configuration and *ci* (*t*) represents its relative contribution to the evolving neural state. Importantly, this decomposition is introduced as a theoretical mathematical representation rather than a directly measured physiological quantity. The present study does not identify individual neural configurations or estimate their relative contributions from the EEG recordings. Instead, the formulation provides a structured mathematical language for describing how multiple distributed neural processes may coexist within a continuously evolving neural state.

One consequence of this representation is that it naturally motivates the integration of multiple complementary electrophysiological measures rather than reliance on any single EEG metric. Signal amplitude characterizes the magnitude of coordinated neural activity, entropy reflects aspects of signal complexity and predictability, oscillatory analyses describe spectral organization, phase-amplitude coupling characterizes interactions across temporal scales, and recurrence quantification analysis evaluates deterministic structure within reconstructed neural trajectories (Richman and Moorman, 2000; Bandt and Pompe, 2002; Tort et al., 2010; Marwan et al., 2007). Within the proposed framework, these observables are not viewed as independent descriptions of neural activity but as complementary mathematical descriptors of the same evolving neural state.

The seizure analyses presented in this dissertation illustrate this principle. Rather than exhibiting isolated changes in a single electrophysiological property, ictal recordings demonstrated coordinated alterations across amplitude, entropy, oscillatory organization, phase dynamics, cross-frequency coupling, and recurrence structure. These observations are consistent with the interpretation that neural dynamics evolve through coordinated changes across multiple measurable dimensions. Importantly, the present findings do not establish the existence of the proposed neural configurations or validate the mathematical decomposition introduced above. Instead, they demonstrate that multiple established electrophysiological observables change systematically during seizure progression and therefore provide an empirical basis for evaluating whether a unified mathematical representation of these dynamics is useful.

The mathematical structure of the proposed framework is loosely inspired by Everett’s relative-state formulation of quantum mechanics (Everett, 1957; DeWitt and Graham, 1973). The analogy is intentionally limited to mathematical structure. Everett’s formalism demonstrates how multiple evolving configurations may be represented within a common state space without requiring wave-function collapse. Likewise, the present framework adopts a similar mathematical perspective for representing multiple simultaneously evolving neural configurations within a single evolving neural state. This comparison should not be interpreted as evidence for quantum computation in the brain, many-worlds ontology, or quantum branching during cognition. Rather, wave-function formalism is employed because it provides a well-developed mathematical language for representing evolving state spaces composed of multiple interacting components.

This perspective also distinguishes the present framework from existing theoretical accounts of consciousness. Integrated Information Theory seeks to quantify the degree of information integration within a system through the scalar quantity Φ (Tononi, 2004; Tononi et al., 2016; Albantakis et al., 2023), whereas electromagnetic field theories identify the brain’s physical electromagnetic field as the substrate of conscious experience (McFadden, 2002, 2020; Pockett, 2000, 2012). By contrast, the present framework does not introduce a new measure of consciousness, propose a new physical mechanism, or identify a specific ontological substrate for conscious experience. Instead, it investigates whether wave-function formalism provides a useful mathematical language for representing the temporal evolution of neural dynamics measured using EEG. The contribution of the present framework is therefore representational rather than mechanistic.

Importantly, the present study does not claim to experimentally validate this mathematical framework. The empirical analyses demonstrate coordinated changes across multiple established electrophysiological observables during seizure progression, but they do not directly identify candidate neural configurations, measure subjective experience, or distinguish conscious from unconscious neural processing. Instead, the proposed formalism generates a research program for future investigation. Multichannel EEG, magnetoencephalography, state-space reconstruction, computational modeling, and paradigms directly comparing conscious and unconscious processing may permit more direct evaluation of whether candidate neural configurations can be identified and whether wave-function formalism provides explanatory advantages over existing descriptions of neural dynamics.

Taken together, the present framework integrates multiple established electrophysiological observables within a common mathematical representation of evolving neural dynamics. Rather than proposing a new electrophysiological measure or a demonstrated theory of consciousness, the present work investigates whether wave-function formalism offers a useful state-space formalism for describing how complementary EEG observables evolve through time. Whether this mathematical framework ultimately provides new insight into unified thought or conscious experience remains an open empirical question, but the present results establish a quantitative foundation upon which that question can be investigated in future work.

### 5.7 Strengths and limitations

The present study possesses several strengths. First, it integrates multiple complementary electrophysiological analyses including signal amplitude, oscillatory organization, entropy, phase dynamics, cross-frequency coupling, and recurrence quantification within a single analytical framework. Rather than relying upon any individual EEG measure, the study characterizes seizure-related neural dynamics across several complementary dimensions of neural organization. This comprehensive analytical approach provides a richer description of evolving neural dynamics, than conventional single-metric analyses while remaining grounded in established computational neuroscience methods. The use of reproducible computational procedures and publicly available EEG datasets further enhances the transparency and reproducibility of the work.

A second strength lies in the deliberate separation between empirical observation and theoretical interpretation. The electrophysiological analyses presented in Chapters 3 and 4 consist entirely of established quantitative EEG methods. The proposed dynamical neural-field framework is introduced only after these empirical findings have been established and is presented as one possible mathematical interpretation of the observed neural dynamics rather than as a direct consequence of the data.

Despite these strengths, several limitations should be acknowledged. First, the empirical analyses were performed using EEG recordings obtained from individuals with epilepsy. Although seizure transitions provide a valuable model for investigating evolving neural dynamics, pathological brain states cannot be assumed to represent the organization of healthy cognition or unified thought. Consequently, the present findings should be interpreted as observations of seizure-related neural dynamics rather than direct evidence regarding normal conscious processing. Future studies incorporating healthy participants, cognitive task paradigms, sleep, anesthesia, and disorders of consciousness will be necessary to determine the broader applicability of the proposed framework.

Second, the EEG recordings analyzed in the present study were obtained from single-channel recordings. While these data provide excellent temporal resolution for examining the evolution of local neural dynamics, they cannot directly quantify large-scale functional connectivity, distributed cortical synchronization, or whole-brain network organization. Accordingly, the present analyses do not directly measure the large-scale neural interactions proposed by the theoretical framework. Future investigations employing high-density EEG, magnetoencephalography, intracranial recordings, and multimodal neuroimaging will be essential for evaluating the spatial organization of the proposed neural-field dynamics.

A further limitation concerns the proposed neural coordination framework itself. The mathematical formulation developed in this dissertation introduces concepts such as simultaneously active neural configurations and transient neural-field dominance as theoretical constructs. Neither the individual neural configurations nor the proposed neural coordination functional,

$$C_{i}(t)=f(A_{i},\phi_{i},H_{i},P,A,C_{i}\mathrm{RQ}A_{i}\ldots )$$

are directly estimated from the present EEG recordings. Instead, the empirical analyses quantify the measurable electrophysiological observables that motivate these theoretical constructs. Consequently, the proposed mechanism should be regarded as a conceptual model that generates experimentally testable hypotheses rather than as an empirically validated description of neural computation.

The mathematical framework is likewise subject to important interpretive limitations. Although the formalism draws mathematical inspiration from Everett’s relative-state representation, the present study does not investigate quantum-mechanical processes in the brain, propose quantum computation, or suggest that neural activity undergoes wave-function collapse or branching into multiple physical realities. Everett’s formulation is employed solely as a mathematical analogy illustrating how multiple simultaneously active neural configurations may be represented within a common dynamical state space. Likewise, the proposed neural field should not be interpreted as the physical electromagnetic field of the brain or as evidence supporting electromagnetic field theories of consciousness. Instead, it serves as a mathematical representation of measurable neural dynamics inferred from electrophysiological observations.

Finally, the present study does not directly measure subjective experience, unified thought, integrated information, metastable neural configurations, or transitions among candidate neural configurations. Instead, it quantifies multiple established electrophysiological observables that describe the temporal organization of evolving neural dynamics. Accordingly, the principal contribution of this dissertation is not the validation of a new theory of consciousness, but the proposal that wave-function formalism provides a useful mathematical framework for representing complementary EEG observables within a common state-space description. Whether this representation ultimately offers explanatory advantages for understanding cognition or consciousness remains an open empirical question.

Taken together, these strengths and limitations position the present work as an initial mathematical framework and research program rather than a definitive account of consciousness or unified thought. The empirical findings establish systematic changes across multiple complementary electrophysiological measures during seizure progression, while the proposed formalism generates explicit hypotheses that can be evaluated in future studies using multichannel electrophysiology, computational state-space reconstruction, and paradigms directly comparing conscious and unconscious neural processing.

### 5.8 Future directions

The present study provides an initial quantitative framework for describing large-scale neural dynamics using complementary electrophysiological measures. Although the current analyses focused on epileptic EEG recordings, the proposed neural-field framework is intended to generate experimentally testable predictions that extend beyond seizure activity. Future investigations should therefore evaluate whether the relationships observed in the present study generalize across healthy cognition, altered states of consciousness, and additional neurological disorders. One important direction for future research is the application of the proposed framework to healthy cognitive processes. Resting-state EEG, task-based paradigms, sensory processing, attention, memory, and decision-making provide opportunities to determine whether coordinated changes in amplitude, entropy, phase organization, cross-frequency coupling, and recurrence dynamics also characterize normal cognitive function. If the proposed framework is broadly applicable, similar patterns of coordinated neural organization should be observable during transitions between cognitive states rather than being limited to pathological neural activity.

The framework may also be evaluated across a wider range of altered states of consciousness. Sleep, general anesthesia, meditation, psychedelic states, and disorders of consciousness each involve measurable changes in large-scale neural dynamics and have been investigated extensively using EEG and magnetoencephalography (MEG). Applying the present analytical pipeline across these conditions would permit direct comparison of dynamical organization across diverse conscious states and determine whether common principles of large-scale neural coordination emerge despite differing physiological mechanisms (Deco et al., 2011; Kelso, 2012).

An additional priority is the incorporation of multimodal neuroimaging approaches. Although EEG provides excellent temporal resolution, combining EEG with MEG, functional magnetic resonance imaging (fMRI), or intracranial EEG could improve characterization of both the temporal and spatial organization of neural activity. Such multimodal approaches may help determine how the reconstructed neural-field dynamics described here relate to underlying structural and functional brain networks (Breakspear, 2017). Future work should also extend the dynamical analyses employed in the present study. The recurrence quantification methods used here provide one description of reconstructed state-space organization, but alternative approaches, including hidden Markov models, neural manifold analysis, dynamical systems modeling, graph-theoretical methods, and nonlinear attractor reconstruction, may provide complementary descriptions of evolving neural activity. Comparing these approaches may clarify which mathematical representations most accurately characterize transitions among large-scale neural configurations.

The proposed framework additionally generates several empirically testable predictions. If unified thought emerges through transient neural-field dominance among multiple simultaneously active neural configurations, future studies should observe systematic relationships between reconstructed state-space geometry, recurrence structure, cross-frequency coordination, and transitions between cognitive states. Similarly, pathological neurological conditions may exhibit reduced diversity of accessible neural configurations together with increased persistence of restricted dynamical states. These predictions can be evaluated directly using longitudinal EEG recordings and large publicly available neurophysiological datasets (Kelso, 2012; Tognoli and Kelso, 2014). An important extension of the present work involves computational modeling. The neural-field equations proposed here may be used to simulate transitions among competing neural trajectories and compared quantitatively with experimentally observed EEG dynamics. Such simulations would permit formal evaluation of whether the proposed mathematical framework reproduces empirical measures of amplitude, entropy, phase organization, cross-frequency coupling, and recurrence dynamics across diverse cognitive conditions. Agreement between simulated and observed neural dynamics would strengthen the explanatory value of the proposed framework while identifying areas requiring refinement.

Finally, the proposed framework may have implications extending beyond neuroscience. Modern machine learning and artificial intelligence increasingly rely upon dynamical representations of high-dimensional state spaces rather than static symbolic computation. Investigating whether principles of metastability, competing neural trajectories, and transient dynamical stabilization improve computational models of adaptive information processing may provide a useful bridge between theoretical neuroscience and biologically inspired artificial intelligence (Friston, 2010; Deco et al., 2011). Although these applications remain speculative, they illustrate the broader potential of viewing cognition as the evolution of coordinated dynamical systems rather than sequences of isolated computational events.

Ultimately, the significance of the proposed framework will depend not on its mathematical elegance but on its capacity to generate falsifiable predictions regarding the organization of large-scale neural dynamics. The present work therefore should be viewed as an initial dynamical framework whose long-term value will be determined by its ability to explain, predict, and experimentally distinguish patterns of unified thought across healthy cognition, altered states of consciousness, and neurological disease.

## 6 Conclusion

The present study investigated whether large-scale neural dynamics observed during epileptic seizure transitions could be interpreted within a dynamical neural-field framework. Using established quantitative EEG analyses, systematic differences were identified across interictal, preictal, and ictal brain states in signal amplitude, oscillatory organization, entropy, phase dynamics, cross-frequency coupling, and recurrence characteristics. Although each analytical approach quantified a distinct aspect of neural activity, the convergence of these complementary findings demonstrated that seizure progression is characterized by coordinated alterations extending across multiple dimensions of evolving neural dynamics measured using EEG.

Importantly, the empirical analyses presented in this dissertation do not directly measure unified thought or consciousness. Rather, they quantify objective properties of evolving neural dynamics measured using EEG, using well-established electrophysiological methods. Collectively, these findings support the view that neural activity is more appropriately described as an evolving dynamical system than as a collection of isolated electrophysiological events. The coordinated changes observed across multiple complementary EEG measures suggest that understanding brain function requires consideration of how distributed neural activity evolves through time rather than reliance upon any single electrophysiological metric.

Building upon these empirical observations, this dissertation proposes that wave-function formalism provides a useful mathematical framework for representing evolving neural dynamics measured using EEG. Within this framework, the neural signal is conceptualized as an evolving state composed of multiple simultaneously active neural configurations, providing a common mathematical representation for complementary electrophysiological observables such as amplitude, phase organization, entropy, cross-frequency coupling, and recurrence structure. The proposed framework does not claim that these neural configurations have been directly identified or that the present findings establish a mechanism of consciousness. Rather, it offers a mathematically grounded representation that generates experimentally testable hypotheses regarding the organization and evolution of neural dynamics, leaving its broader implications for cognition and conscious experience to be evaluated in future investigations.

The principal contribution of the present work is therefore not the introduction of a new electrophysiological measure or a new ontological theory of consciousness. Instead, it proposes a mathematically coherent and experimentally testable framework for interpreting multiple established electrophysiological observables within a single dynamical description of neural organization. In doing so, the framework provides a proposed mechanism through which one unified thought may emerge from multiple simultaneously active neural configurations while remaining compatible with existing theories of large-scale neural organization. The present framework should therefore be viewed as complementary to existing approaches rather than competitive with them. Integrated Information Theory emphasizes the degree of information integration within neural systems, whereas electromagnetic field theories focus on the possible causal role of the brain’s electromagnetic field.

Ultimately, the significance of the proposed framework will depend not on its mathematical elegance alone, but on its empirical utility. Its value will be determined by whether wave-function formalism provides explanatory or predictive advantages over existing mathematical descriptions of neural dynamics and whether it generates experimentally distinguishable predictions that can be validated using multichannel electrophysiology, computational modeling, and paradigms directly comparing conscious and unconscious neural processing. The present work should therefore be viewed as the beginning of a research program aimed at evaluating the usefulness of wave-function formalism as a mathematical representation of evolving neural dynamics. Future work should seek not only to validate the proposed mathematical framework, but also to identify the conditions under which it fails. Establishing clear criteria for falsification will be essential for determining whether wave-function formalism offers genuine explanatory value beyond existing dynamical systems approaches.

## Data Availability

The original contributions presented in the study are included in the article/supplementary material, further inquiries can be directed to the corresponding author.
